# A phosphotyrosine switch regulates organic cation transporters

**DOI:** 10.1038/ncomms10880

**Published:** 2016-03-16

**Authors:** Jason A. Sprowl, Su Sien Ong, Alice A. Gibson, Shuiying Hu, Guoqing Du, Wenwei Lin, Lie Li, Shashank Bharill, Rachel A. Ness, Adrian Stecula, Steven M. Offer, Robert B. Diasio, Anne T. Nies, Matthias Schwab, Guido Cavaletti, Eberhard Schlatter, Giuliano Ciarimboli, Jan H. M. Schellens, Ehud Y. Isacoff, Andrej Sali, Taosheng Chen, Sharyn D. Baker, Alex Sparreboom, Navjotsingh Pabla

**Affiliations:** 1 Department of Pharmaceutical, Social and Administrative Sciences, School of Pharmacy, D'Youville College, Buffalo, New York 14201, USA; 2 Department of Chemical Biology & Therapeutics, St Jude Children's Research Hospital, Memphis, Tennessee 38105, USA; 3 Division of Pharmaceutics, College of Pharmacy & Comprehensive Cancer Center, The Ohio State University, Columbus, Ohio 43210, USA; 4 Department of Oncology, St. Jude Children's Research Hospital, Memphis, Tennessee 38105, USA; 5 Department of Pharmaceutical Sciences, St Jude Children's Research Hospital, Memphis, Tennessee 38105, USA; 6 Department of Molecular and Cell Biology, University of California, Berkeley, California 94720, USA; 7 Department of Bioengineering and Therapeutic Sciences, University of California, San Francisco, California 94158, USA; 8 Department of Molecular Pharmacology and Experimental Therapeutics, Mayo Clinic Cancer Center, Rochester, Minnesota 55905, USA; 9 Dr Margarete Fischer-Bosch Institute of Clinical Pharmacology, 70376 Stuttgart, Germany; 10 University of Tuebingen, 72074 Tuebingen, Germany; 11 Department of Clinical Pharmacology, University Hospital, 72076 Tuebingen, Germany; 12 Department of Surgery and Translational Medicine, University of Milano-Bicocca, 20900 Monza, Italy; 13 Medical Clinic D, Experimental Nephrology and Interdisciplinary Center for Clinical Research (IZKF), Münster Medical Faculty, 48149 Münster, Germany; 14 Division of Molecular Pathology, The Netherlands Cancer Institute, 1066 CX Amsterdam, The Netherlands; 15 California Institute for Quantitative Biosciences, University of California, San Franscisco, California 94720, USA; 16 Department of Pharmaceutical Chemistry, University of California, San Franscisco, California 94158, USA

## Abstract

Membrane transporters are key determinants of therapeutic outcomes. They regulate systemic and cellular drug levels influencing efficacy as well as toxicities. Here we report a unique phosphorylation-dependent interaction between drug transporters and tyrosine kinase inhibitors (TKIs), which has uncovered widespread phosphotyrosine-mediated regulation of drug transporters. We initially found that organic cation transporters (OCTs), uptake carriers of metformin and oxaliplatin, were inhibited by several clinically used TKIs. Mechanistic studies showed that these TKIs inhibit the Src family kinase Yes1, which was found to be essential for OCT2 tyrosine phosphorylation and function. Yes1 inhibition *in vivo* diminished OCT2 activity, significantly mitigating oxaliplatin-induced acute sensory neuropathy. Along with OCT2, other SLC-family drug transporters are potentially part of an extensive ‘transporter-phosphoproteome' with unique susceptibility to TKIs. On the basis of these findings we propose that TKIs, an important and rapidly expanding class of therapeutics, can functionally modulate pharmacologically important proteins by inhibiting protein kinases essential for their post-translational regulation.

## Abstract (web summary)

Organic cation transporters are important drug transporters that influence therapeutic outcomes. Here, the authors find that these transporters are regulated by tyrosine phosphorylation and propose that tyrosine kinase inhibitors can influence drug transporter function through post-translational mechanisms.

## Graphical abstract

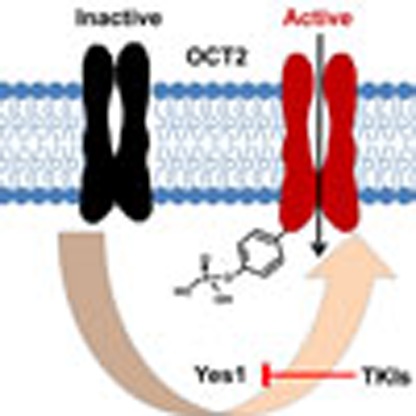

An enclosed but selectively permeable cellular membrane is a ubiquitous feature of all life forms^1^,^2^. Membrane transporters are the evolutionally conserved gatekeepers that govern this selective cellular permeability^3^. They regulate the uptake and efflux of essential molecules such as amino acids, nucleosides, sugars, inorganic ions as well as therapeutic compounds^3^,^4^. Therefore, transporters have wide ranging influences on normal human physiology and pathophysiology and are key determinants of therapeutic response to drugs.

The human genome is thought to encode more than 400 membrane transporter genes belonging to two major superfamilies: ATP-binding cassette transporters and solute carriers (SLC), which are involved in almost every essential biological process^4^,^5^,^6^,^7^. Among these, about 20 ‘multispecific' transporters belonging to either superfamily have been extensively implicated in drug transport^6^,^8^,^9^. Drug transporters are highly expressed in the intestine, kidney, liver and endothelial barriers, where they regulate absorption, distribution, metabolism and excretion of drugs^6^,^8^. At the cellular level, transporter-mediated uptake or efflux can impart drug-sensitive or -resistant^10^ phenotypes in target cells, thereby affecting therapeutic efficacy. Likewise, transporter-mediated uptake in non-target tissues can contribute to drug toxicities^9^,^11^. As a result, along with drug-metabolizing enzymes, transporters have emerged as critical determinants of drug disposition, therapeutic efficacy and adverse drug reactions.

Due to their predominant role in determining clinical response to therapeutics, multiple regulatory aspects of drug transporters have been widely studied. Genetic polymorphisms^12^, epigenetic mechanisms^13^, dietary ingredients^14^ and drug–drug interactions^6^ that functionally modulate drug transporters can profoundly affect therapeutic outcomes. However, surprisingly, phosphotyrosine-mediated regulation of drug transporters has not been extensively studied. Here we report phosphotyrosine-mediated regulation of several pharmacologically important SLC-family transporters, including organic cation transporters (OCTs)^15^, multidrug and toxin-extrusion transporters (MATEs)^16^ and organic anion transporting polypeptides (OATPs)^17^. Notably, we propose that clinically used tyrosine kinase inhibitors (TKIs)^18^ can inhibit protein kinases required for tyrosine phosphorylation of drug transporters, thereby influencing transporter function.

## Results

### Small-molecule screen identifies potent OCT2 inhibitors

**Figure 1:**
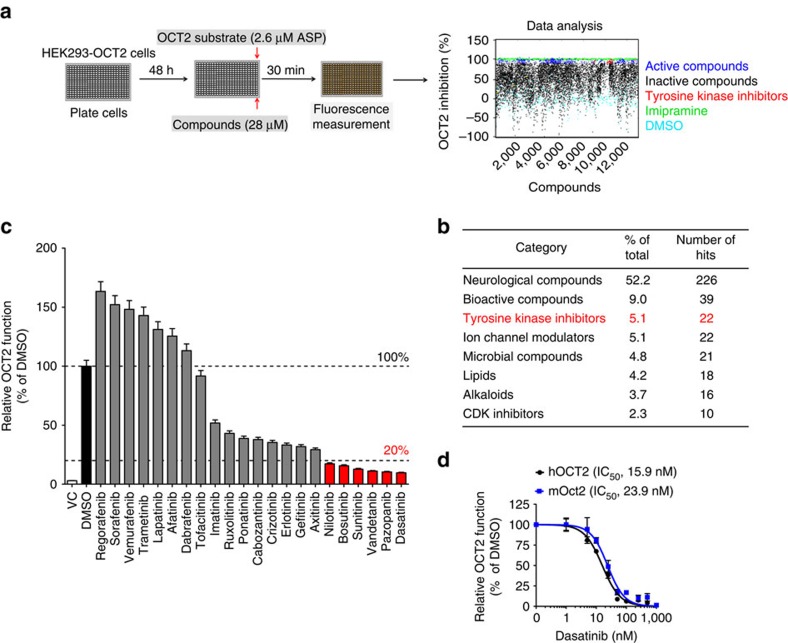
Small molecule HTS of OCT2 inhibitors. (**a**) Scheme depicting the assay conditions used in the primary screen for OCT2 inhibitors. HEK293-OCT2 cells were plated in 384-well plates, followed by incubation with small molecules and uptake assay using ASP as OCT2 substrate. Imipramine and DMSO were used as positive and negative controls respectively. Compounds that inhibited OCT2 activity by ≥90% were considered as active compounds. (**b**) Active compounds (433 out of 8,086) were categorized into distinct groups and the categories with more than 2% hits are shown here. (**c**) Secondary screens were carried out using Hela-OCT2 cells. These cells were preincubated with 10 μM concentration of TKIs for 15 min, followed by incubation with OCT2 substrate [^14^C]-TEA. Data are presented as percentage OCT2 activity (TEA uptake) as compared to DMSO group. (**d**) HEK293-hOCT2 and HEK293-mOct2 cells were preincubated with varying concentrations of dasatinib for 15 min, followed by incubation with 2 μM [^14^C]-oxaliplatin for 15 min. Data are presented as percentage OCT2 activity (oxaliplatin uptake) as compared with DMSO group. All experimental values are presented as mean±s.e.m. The height of error bar=1 s.e.

The SLC-family member, OCT2, is expressed in the renal tubular cells^15^, dorsal root ganglia (DRG)^19^ and brain^20^, where it regulates uptake of endogenous organic cations like creatinine^21^ and catecholamines^22^. OCT2 is also involved in the uptake of widely used therapeutics including the anti-diabetic drug metformin^23^ and platinum-based chemotherapeutics^19^,^24^. The three major debilitating side effects of platinum-based anti-cancer drugs, namely peripheral neurotoxicity^19^, nephrotoxicity^24^,^25^ and ototoxicity^26^, are dependent on OCT2-mediated uptake of cisplatin or oxaliplatin in DRGs, renal tubular cells and hair cells of the cochlea, respectively. Identification of potent OCT2 inhibitors, which could be combined with chemotherapy, has the potential to provide clinical benefits by reducing these toxicities. Thus, with the aim to identify OCT2 inhibitors, we carried out a small-molecule screen using the St Jude Children's Research Hospital bioactive compound library (8086 compounds). For this screen, we utilized OCT2 expressing HEK293 cells and uptake of fluorescent substrate 4-4-[4-(dimethylamino)styryl]-*N*-methylpyridinium (ASP) was used as an indicator of OCT2 function (Fig. 1a). Imipramine, a known OCT2 inhibitor, was used as a positive control (Fig. 1a). We identified 433 compounds that exhibited >90% inhibition of OCT2 function (Fig. 1b and Supplementary File 1). About half of these active compounds were neurological drugs, which was not surprising because they are structurally similar to endogenous OCT2 substrates like norepinephrine and serotonin^20^. Due to their undesirable neurological effects these agents were not explored further.

Multiple TKIs also inhibited OCT2 function, and since they are widely used as anti-cancer agents^18^ we pursued them further (Fig. 1b). FDA-approved TKIs were tested for their ability to inhibit OCT2 in secondary screens using tetraethylammonium (TEA) as OCT2 substrate (Fig. 1c). Dose–response experiments in OCT2 expressing Hela (Hela-OCT2) and HEK293 (HEK293-OCT2) cells showed that multiple TKIs can inhibit OCT2 function at sub-micromolar concentrations (Supplementary Fig. 1a). Creatinine is a OCT2 substrate and interestingly, the TKIs (bosutinib, dasatinib, nilotinib, pazoponib, sunitinib and vandetinib) that inhibited OCT2 function *in vitro* can cause increase in serum creatinine levels (Supplementary Fig. 1b) in patients, which could be a result of reduced creatinine excretion as a result of renal OCT2 inhibition. Among these TKIs, dasatinib^18^,^27^, an oral Bcr-Abl and Src-family kinase inhibitor approved for the treatment of leukaemia, was found to be the most potent OCT2 inhibitor (Fig. 1c), with OCT2 inhibition seen at nanomolar concentrations (Fig. 1d).

We next considered if TKIs inhibited OCT2 through a competitive mechanism. We found that OCT2 inhibiting TKIs, including dasatinib were not OCT2 substrates (Supplementary Fig. 1c) and dasatinib-mediated OCT2 inhibition was found to be non-competitive (Supplementary Fig. 1d). In addition, dasatinib-mediated OCT2 inhibition was found to be reversible (Supplementary Fig. 1e). A previous study^28^ also reported dasatinib-mediated OCT2 inhibition albeit with significantly less potency. In that study^28^, the substrates and inhibitors were coincubated, while we preincubated cells with dasatinib. Indeed, preincubation with TKIs was sufficient and, in fact, more effective in inhibiting OCT2 function than co-incubation (Supplementary Fig. 1f).

### Tyrosine phosphorylation is essential for OCT2 function

**Figure 2:**
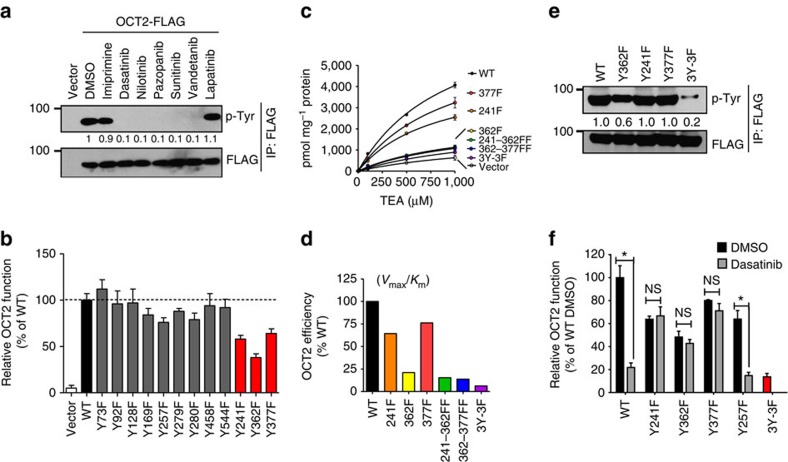
Functional regulation of OCT2 by tyrosine phosphorylation. (**a**) Hela-Vector or Hela-OCT2 cells were treated with either DMSO, TKIs (1 μM) or 500 μM imipramine for 30 min. Cell lystates were then used for immunoprecipitation of FLAG-OCT2 with mouse anti-FLAG antibodies, followed by western blot analysis with rabbit phospho-tyrosine and FLAG antibodies. (**b**) Plasmids for OCT2 mutants were transiently transfected into Hela cells and 24 h later, uptake assays (15 min) were performed using [^14^C]-TEA (2 μM). TEA uptake levels were normalized to protein levels in each group. The graph represents relative OCT2 function (TEA uptake) as compared with wild-type OCT2 transfected group. (**c**,**d**) Plasmids for indicated OCT2 mutants were transiently transfected into Hela cells and 24 h later, uptake assays were performed using varying concentration of [^14^C]-TEA. TEA uptake levels were normalized to protein levels in each group and graphs represent Michaelis–Menten kinetics and calculated ratio of *K*_m_/*V*_max_ values, which are indicative of transporter activity. (**e**) Hela cells were transiently transfected with indicated FLAG-tagged OCT2 constructs, followed by immunopreicipation with anti-FLAG bead conjugated antibodies and western blot analysis by FLAG and phosphotyrosine antibodies. (**f**) Hela cells were transiently transfected with indicated plasmids, followed by TEA uptake assays in the presence of DMSO or 1 μM dasatinib. * indicates statistically significant as compared to respective DMSO treated group (Student's *t*-test). All experimental values are presented as mean±s.e.m. The height of error bar=1 s.e.

To gain further mechanistic insights, we performed surface biotinylation assays and found that TKIs did not affect membrane OCT2 expression (Supplementary Fig. 2a). Although tyrosine phosphorylation of OCTs has not been studied, we considered the possibility that TKIs might inhibit OCT2 function by modulating its tyrosine phosphorylation. Indeed, immunoprecipitation (IP) of FLAG-OCT2 from Hela-OCT2 cells showed that OCT2 was tyrosine phosphorylated (Fig. 2a). Importantly, OCT2-inhibiting TKIs (dasatinib, nilotinib, pazoponib, sunitinib and vandetinib) also inhibited its tyrosine phosphorylation. This was not observed for lapatinib, a TKI that did not inhibit OCT2 as well as for imipramine, a competitive OCT2 inhibitor (Fig. 2a). Tyrosine phosphorylation of OCT2 was further corroborated by reverse-IP experiments, where we immunoprecipitated total tyrosine phosphorylated proteins using a phosphotyrosine antibody and could pull-down FLAG-OCT2 (Supplementary Fig. 2b). Moreover, tyrosine phosphorylation of OCT2 was inhibited by dasatinib in a time-dependent manner, which indicated that OCT2 tyrosine phosphorylation could be a dynamic process (Supplementary Fig. 2c).

To identify the phosphotyrosine sites in OCT2 we utilized computational prediction tools^29^ and PhosphoSite^30^,^31^, a database of post-translational modifications derived from discovery-mode tandem mass spectrometry (MS) experiments. Subsequently, functional assays of several tyrosine-to-phenylalanine OCT2 mutants showed that mutations at three sites (241, 362 and 377) substantially reduced OCT2 function, without affecting membrane OCT2 expression (Fig. 2b; Supplementary Fig. 2d,e). These three sites were originally selected on the basis of MS data in PhosphoSite^30^,^31^, which showed that the Y241 site in OCT2 is tyrosine phosphorylated while sites corresponding to OCT2 Y362 and Y377 are tyrosine phosphorylated in the closely related transporters OCT1 and OCTN2. Interestingly, tyrosine-to-phenylalanine mutations at computationally predicted tyrosine sites had no effect on OCT2 function, while all the three sites for which MS data exist in Phosphosite were found to be important for OCT2 function. Further kinetic experiments showed that the three mutants had reduced OCT2 function, with Y362F mutation having the most dramatic effect (Fig. 2c,d). Immunoprecipitation experiments showed that the Y362F mutant had significantly reduced tyrosine phosphorylation, which was further diminished in the triple-mutant (Y241F-Y362F-Y377F, denoted as 3Y–3F; Fig. 2e). These three sites are evolutionarily conserved (Supplementary Fig. 2f). While these OCT2 mutants had lower baseline function, they were resistant to dasatinib-mediated OCT2 inhibition (Fig. 2f).

To understand how post-translational modifications affect OCT2 function, we considered the possibility that tyrosine phosphorylation may regulate OCT2 oligomerization. However, single-molecule subunit analysis^32^,^33^ experiments showed that OCT2 most likely exists as a monomer (Supplementary Fig. 3a-c). Although the crystal structure of OCT2 is not known, previous^34^ OCT1 structural models and new OCT2 modelling data (Supplementary Fig. 3d) suggest that the Y362 site is localized close to the substrate-binding domain. This raises the possibility that the negative charge provided by a phosphotyrosine may enhance substrate binding of positively charged organic cations. However, we cannot rule out the possibility that the Y362F mutant has reduced function due to diminished substrate binding, but the presence of MS data^30^ showing tyrosine phosphorylation at this site in OCT1 combined with our results that Y362F OCT2 mutant has reduced tyrosine phosphorylation and is resistant to dasatinib-mediated OCT2 inhibition suggests that the reduced function of Y362F mutant could be due to decreased tyrosine phosphorylation.

### siRNA screen identifies Yes1 as OCT2 phosphorylating kinase

**Figure 3:**
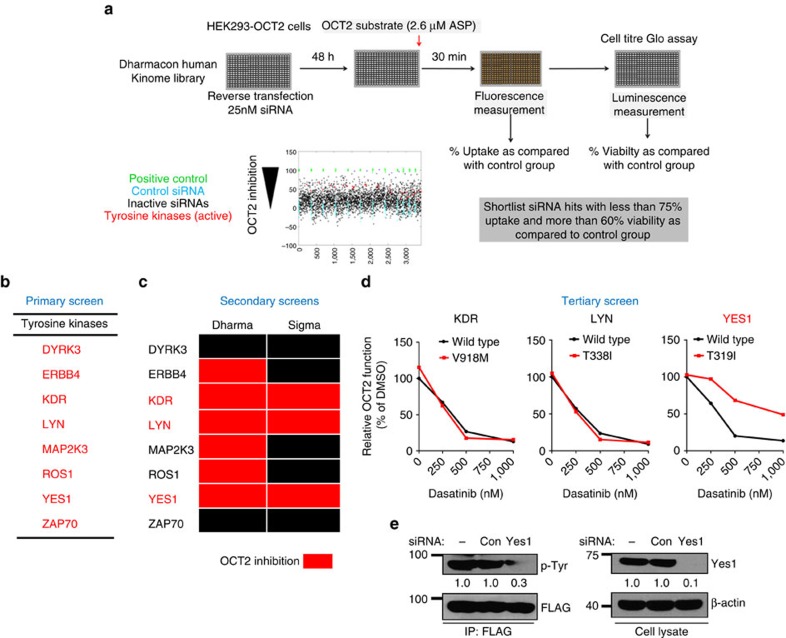
Functional regulation of OCT2 by tyrosine phosphorylation. (**a**) Scheme depicting the assay conditions used in the primary siRNA kinome screen to identify an OCT2 phosphorylating kinase. HEK293-OCT2 cells were reverse transfected with the siRNA library and plated in 384-well plates, followed by incubation functional uptake and viability assays. (**b**) Positive hits from the primary screen. Only the tyrosine kinase hits (indicated in red) were used for secondary screens. (**c**) Schematic representation of secondary screens: deconvoluted screen using Dharmacon siRNA and Sigma siRNA screen. The siRNA that inhibited OCT2 function to at least 75% are indicated in red. (**d**) Hela-OCT2 cells were transfected with either wild-type or dasatinib resistant KDR, LYN or Yes1 plasmids and 24 h later, [^14^C]-TEA uptake assays were performed in the presence or absence of varying concentrations of dasatinib. The graph represents relative OCT2 function ([^14^C]-TEA uptake) as compared with DMSO group for each plasmid. (**e**) Hela-OCT2 cells were transfected with Sigma siRNA (scrambled control or Yes1) and 48 h later, OCT2 was immunoprecipitated to determine its tyrosine phosphorylation. Whole-cell lysate was used to confirm Yes1 knockdown.

To identify upstream kinases involved in OCT2 phosphorylation, we performed a primary siRNA screen using a human protein kinase siRNA library (779 genes; Dharmacon) to knockdown protein kinases in HEK293-OCT2 cells followed by functional assays (Fig. 3a). The siRNAs (tyrosine kinases) that reduced OCT2 function to ≤75% without affecting cell viability were selected for further consideration (Fig. 3b; Supplementary File 2). Next, we performed a deconvolution secondary screen with ASP as OCT2 substrate (Fig. 3c) and an additional secondary screen utilizing pooled siRNAs (Sigma) with TEA as OCT2 substrate (Fig. 3c; Supplementary Fig. 4). On the basis of the positive hits from both these secondary screens, we narrowed down the putative targets to KDR, LYN and Yes1 (Fig. 3c). To unambiguously identify the TKI-sensitive protein kinase that phosphorylates OCT2, we carried out tertiary screens utilizing a chemical genetics approach^35^. The TKI resistance of gatekeeper mutants of KDR, LYN and Yes1 was initially confirmed (Supplementary Fig. 5a). We then transfected Hela-OCT2 cells with either the wild-type or TKI-resistant (gatekeeper) mutants^35^ of KDR, LYN and Yes1, followed by dasatinib treatment and OCT2 uptake assays. As shown in Fig. 3d, only the TKI-resistant Yes1 mutant was able to rescue OCT2 inhibition by dasatinib. The TKI-resistant Yes1 mutant was also able to significantly rescue OCT2 inhibition by other TKIs (Supplementary Fig. 5b). Moreover, Yes1 knockdown significantly reduced OCT2 tyrosine phosphorylation (Fig. 3e). Indeed, previous^36^ analysis of kinase inhibition selectivity has shown that all the TKIs that inhibited OCT2 function can inhibit Yes1 (Supplementary Fig. 6a). Moreover, other non-TKI Yes1 inhibitors like dorsomorphin^37^ were also found to be potent OCT2 inhibitors (Supplementary Fig. 6b). Importantly, OCT2 phosphotyrosine sites have sequences similar to known Src family kinase substrates (Supplementary Fig. 7a). *In vitro* kinase assays with purified Yes1 and OCT2 proteins showed that Yes1 can phosphorylate OCT2 and this tyrosine phosphorylation is significantly reduced in the Y362F and 3Y–3F mutants (Supplementary Fig. 7b). These data suggest that Yes1 is the TKI-sensitive kinase that can directly phosphorylate OCT2. On the basis of these studies, we propose that these three tyrosines may be phosphorylated by Yes1, but the Y362 might be the major phosphotyrosine site that has greater functional relevance. On the basis of these studies, Yes1 inhibition is likely a key mechanism of dasatinib-mediated OCT2 inhibition, however, the role of other kinases as well as other non-kinase mechanisms cannot be excluded for other TKIs.

### Tyrosine phosphorylation is conserved in OCTs

**Figure 4:**
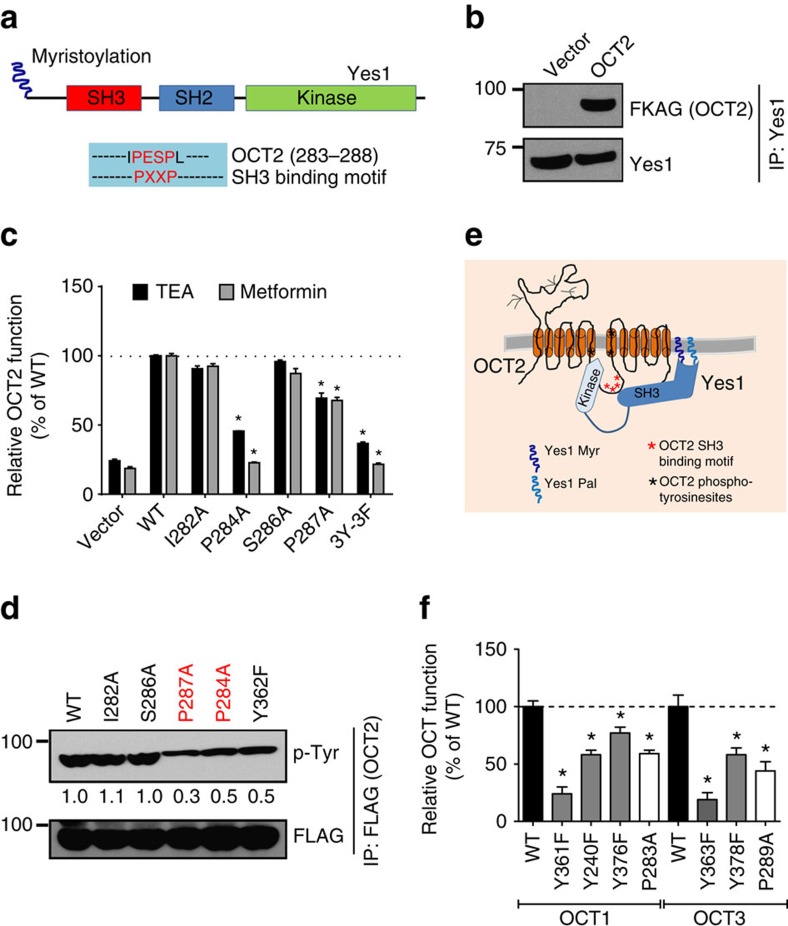
Yes1-mediated regulation of OCT2 tyrosine phosphorylation. (**a**) Schematic representation of Yes1 protein (upper panel) showing the SH3 domain. The lower panel shows the putative proline-rich SH3 binding sequence in OCT2. (**b**) Endogenous Yes1 was immunoprecipitated from Hela-Vector and Hela-OCT2 cell lysates using a mouse anti-Yes1 antibody, followed by western blot analysis with rabbit anti-FLAG and Yes1 antibodies. (**c**) Plasmids for OCT2 mutants were transiently transfected into Hela cells and 24 h later, uptake assays (15 min) were performed using [^14^C]-TEA (2 μM) or [^14^C]-metformin (50 μM). The uptake levels were normalized to protein concentration in each group. The graph represents relative OCT2 function (TEA or metformin uptake) as compared to wild-type OCT2 transfected group. * indicates statistically significant as compared with wild-type group (*P*<0.05, Student's *t*-test). (**d**) Hela cells were transiently transfected with indicated FLAG-tagged OCT2 constructs, followed by immunopreicipation with anti-FLAG antibodies and western blot analysis by FLAG and phosphotyrosine antibodies. (**e**) Proposed model of Yes1 and OCT2 interaction. (**f**) Plasmids for OCT1 and OCT3 mutants were transiently transfected in Hela cells and 24 h later, uptake assays (15 min) were performed using [^14^C]-TEA (2 μM). The uptake levels were normalized to protein concentration and the graph represents relative OCT2 function (TEA uptake) as compared with respective wild-type group. * indicates statistically significant as compared to wild-type group (*P*<0.05, Student's *t*-test). All experimental values are presented as mean±s.e.m. The height of error bar=1 s.e.

While further exploring the OCT2 regulation by Yes1, we noticed that OCT2 has a proline-rich (PXXPR) sequence, which is known to bind Src Homology 3 (SH3)^38^,^39^ domain present in Yes1 (Fig. 4a). Indeed, co-immunoprecipitation experiments showed that Yes1 can physically associate with OCT2 (Fig. 4b), and mutations in the proline-rich SH3-binding domain reduced OCT2 function (Fig. 4c) and tyrosine phosphorylation (Fig. 4d). Although Yes1 is a non-receptor tyrosine kinase, it is both myristoylated and palmitoylated, which allows it to tether to membranes both in the endoplasmic reticulum (ER) and the plasma membrane^40^. Tyrosine phosphorylated OCT2 was present in both the ER and plasma-membrane fractions (Supplementary Fig. 7c) and it is likely that Yes1 might phosphorylate OCT2 in the ER and/or at the plasma membrane (Fig. 4e). The OCT2 phosphotyrosine sites are conserved in the related transporters OCT1 and OCT3, which are also inhibited by dasatinib (Supplementary Fig. 8a,b). The proline-rich SH3-binding sequence is also conserved in OCT1, OCT3, OCTN1 and OCTN2 (Supplementary Fig. 8a). In humans, a naturally occurring single nucleotide variant in the *OCT1* gene, causing a P283L change, is known to reduce OCT1 function^41^. Interestingly, this site is located in the proline-rich SH3 binding sequence of OCT1. To examine whether the phosphorylation-mediated regulation of OCT2 is conserved in OCT1 and OCT3, we carried out functional assays after mutagenesis of relevant sites. As shown in Fig. 4f, OCT1 and OCT3 mutants that lacked either the putative phosphorylation sites or the proline-rich motif had significantly reduced function.

The existence of a conserved regulatory mechanism in OCT1, OCT2, and OCT3 led us to question if other drug transporters are also regulated by tyrosine phosphorylation. Interestingly, MS data from global phosphoproteome studies^30^,^31^ suggest that a substantial fraction of clinically relevant transporters, drug-metabolizing enzymes, and ion channels have sites that are tyrosine phosphorylated (Supplementary File 3; Supplementary Fig. 9). To test whether these phosphotyrosine modifications have a functional role, we generated tyrosine-to-phenylalanine mutants for the SLC-family drug-transporters MATE1 (ref. 16) and OATP1B1 (ref. 17). Mutations at these conserved sites significantly reduced transporter activity, indicating functional relevance (Supplementary Fig. 10a–d). Moreover, distinct TKIs can inhibit these SLC-family drug transporters (Supplementary Fig. 11). These studies suggest that tyrosine phosphorylation may be a potentially widespread mechanism of drug transporter regulation that might be a target of deregulation by clinically used TKIs.

### Yes1-mediated regulation of OCT2 function in the kidney

**Figure 5:**
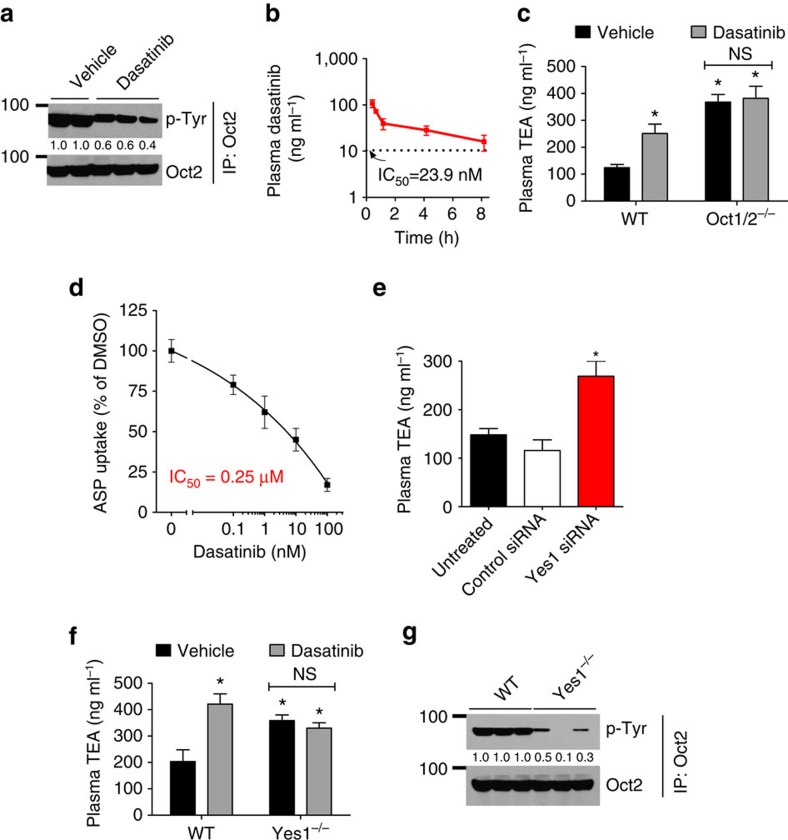
OCT2 tyrosine phosphorylation and functional regulation *in vivo*. (**a**) Wild-type FVB mice were injected with either vehicle or dasatinib (15 mg kg^−1^, p.o.) and 30 min later, the mice were euthanized and the kidneys were collected. Kidney tissue lysates were then used to immunoprecipitate endogenous Oct2 followed by western blot analysis by phosphotyrosine and Oct2 antibodies. (**b**) Male FVB mice were injected with dasatinib (15 mg kg^−1^) followed by pharmacokinetic analysis of dasatinib levels in the plasma. (**c**) Wild-type and Oct1/2^−/−^ mice were injected with either vehicle or dasatinib (15 mg kg^−1^, p.o.) and 30 min later they were injected with 0.2 mg kg^−1^ [^14^C]-TEA (i.v.), followed by plasma collection at 5 min. The graph represents plasma TEA levels from *n*=5 mice per group. * indicates statistically significant as compared with wild-type vehicle group (*P*<0.05, Student's *t*-test). (**d**) Isolated renal tubules were coincubated with dasatinib in the presence of the OCT2 substrate ASP (30 min), and relative uptake was measured compared with control group. (**e**) Wild-type FVB mice were injected with either control or Yes1 siRNA by hydrodynamic tail-vein injection (25 μg in 0.5 ml of PBS). Three days later, the mice were injected i.v. with a 0.2 mg kg^−1^ dose of [^14^C]-TEA, and plasma levels of TEA were measured at 5 min (*n*=5 mice per group). Kidneys were also collected to determine Yes1 knockdown. (**f**) Wild-type and Yes1^−/−^ mice (*n*=5) were injected with 0.2 mg kg^−1^ [^14^C]-TEA (i.v.) and plasma levels of TEA were measured at 5 min. (**g**) Kidney tissue lysates from wild-type and Yes1^−/−^ were used to immunoprecipitate endogenous Oct2 followed by western blot analysis by phosphotyrosine and Oct2 antibodies. All experimental values are presented as mean±s.e.m. The height of error bar=1 s.e.

We next examined whether OCT2 is tyrosine phosphorylated *in vivo*, initially in the kidney, a major site of OCT2 expression^42^. Endogenous OCT2 in murine kidneys was tyrosine phosphorylated, and dasatinib administration significantly inhibited OCT2 tyrosine phosphorylation (Fig. 5a). Dasatinib administration followed by pharmacokinetic analysis showed that plasma dasatinib levels were significantly higher than the concentrations required for inhibiting OCT2 function *in vitro* (Fig. 5b). Next, we utilized a previously established^24^ *in vivo* functional assay where the OCT2 substrate TEA is injected in mice and OCT2 inhibition under these conditions leads to increased plasma TEA levels. In the wild-type mice, dasatinib treatment led to increased plasma TEA levels (Fig. 5c) and reduced urinary TEA excretion (Supplementary Fig. 12a), indicating OCT2 inhibition. Importantly, in the Oct1/2 deficient, dasatinib treatment did not alter the TEA levels in the plasma or urine (Fig. 5c; Supplementary Fig. 12a). Dasatinib-mediated OCT2 inhibition was further confirmed in e*x vivo* renal tubule uptake assays^43^ (Fig. 5d). Next, we used hydrodynamic siRNA injection^44^ to knockdown Yes1 in the kidneys, which resulted in reduced OCT2 phosphorylation (Supplementary Fig. 12b) and diminished OCT2 function (Fig. 5e; Supplementary Fig. 12c). Yes1 knockout mice also had reduced OCT2 function (Fig. 5f; Supplementary Fig. 12d) and lower OCT2 tyrosine phosphorylation (Fig. 5g). The levels of dasatinib in the plasma of WT and Yes1-deficient mice was not significantly different (Supplementary Fig. 12e). These studies using multiple experimental approaches established that Yes1-mediated OCT2 phosphorylation is critical for OCT2 function *in vivo*.

### Yes1 inhibition ameliorates oxaliplatin-induced acute sensory neuropathy

**Figure 6:**
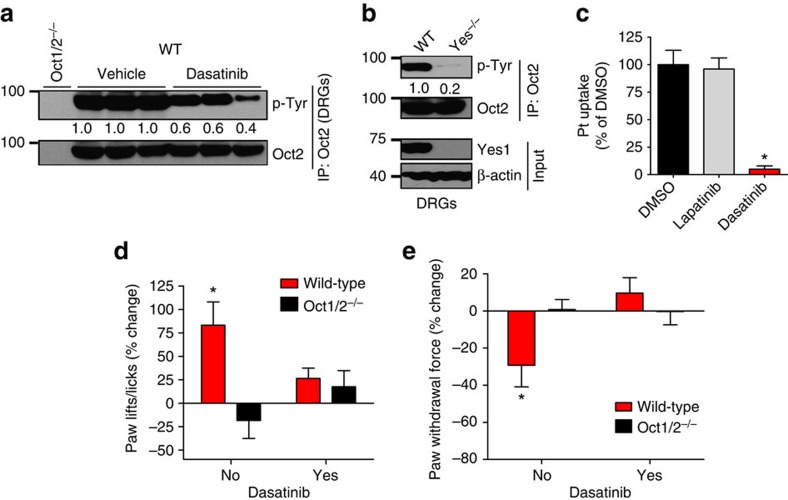
Yes1 inhibition mitigates Oct2-dependent oxaliplatin neurotoxicity. (**a**) Wild-type FVB mice were injected with vehicle and the Oct1/2^−/−^ mice were injected with either vehicle or dasatinib (15 mg kg^−1^, p.o.) and 30 min later, DRGs were collected. DRG lysates were then used to immunoprecipitate endogenous Oct2 followed by western blot analysis by phospho-tyrosine and Oct2 antibodies. (**b**) DRGs were collected from Wild-type and Yes1^−/−^ mice. The upper panel shows representative blots from experiments where DRG lysates were used to immunoprecipitate endogenous Oct2 followed by western blot analysis by phospho-tyrosine and Oct2 antibodies. The lower panel shows western blot results from total DRG lysates showing that Yes1 is expressed in DRGs in the wild-type mice. (**c**) DRGs were collected from Wild-type FVB mice, followed by satellite cell isolation and culture. The primary satellite cells were then plated in six-well plates followed by oxaliplatin uptake assays in the presence of DMSO, lapatinib or Dasatinib (30 min). The graph represents relative oxaliplatin uptake as compared to DMSO group. * indicates a statistically significant difference compared with the DMSO group. (**d**) Sensitivity to cold associated with a single dose of oxaliplatin (40 mg kg^−1^) in wild-type mice pretreated with vehicle or dasatinib (15 mg kg^−1^, p.o.) as determined by a cold-plate test. The number of paw lifts or licks at baseline and following exposure to a temperature of −4 °C for 5 min at 24 h after drug administration was determined (*n*=5). The graph represents relative percentage change in paw lifts/licks as compared with baseline values. (**e**) Mechanical allodynia associated with a single dose of oxaliplatin (40 mg kg^−1^) in wild-type mice pretreated with vehicle or dasatinib (15 mg kg^−1^, p.o.), as determined by a Von Frey Hairs test. The force required to induce paw withdrawal in grams (g) at baseline was measured following 24 h after drug administration (*n*=5). The graph represents relative percentage change in paw withdrawal force as compared to baseline values. * indicates a statistically significant difference as compared with the baseline (untreated) values. All experimental values are presented as mean±s.e.m. The height of error bar=1 s.e.

Finally, we examined whether inhibition of Yes1 can diminish OCT2 function in DRGs and reduce oxaliplatin-induced acute sensory neuropathy^19^. Indeed, dasatinib treatment reduced OCT2 tyrosine phosphorylation in DRGs, the primary site of oxaliplatin uptake^19^ and peripheral neurotoxicity (Fig. 6a). Moreover, DRGs isolated from Yes1-deficient mice had significantly reduced OCT2 tyrosine phosphorylation (Fig. 6b). Since OCT2 is expressed in satellite cells in DRGs^19^, we cultured primary satellite cells from murine DRGs. Uptake assays showed that dasatinib but not lapatinib inhibited oxaliplatin uptake in these cells (Fig. 6c). To assess the therapeutic potential of dasatinib in preventing oxaliplatin-induced acute sensory neuropathy, we performed experiments in previously established mouse models^19^. Dasatinib treatment significantly mitigated both mechanical allodynia and cold sensitivity, markers of oxaliplatin-induced acute sensory neuropathy (Fig. 6d,e), without affecting the systemic disposition of oxaliplatin (Supplementary Fig. 13a,b) or the metabolism and plasma levels of fluoropyrimidines such as fluorouracil^45^, which are commonly given concurrently with oxaliplatin (Supplementary Fig. 13c,d). Furthermore, Yes1-deficient mice were also protected from oxaliplatin-induced acute sensory neuropathy (Supplementary Fig. 14). While we observed that Yes1 inhibition resulted in significant mitigation of oxaliplatin-induced acute sensory neuropathy, the effect of dasatinib on chronic neuropathy is currently unknown. However, recent studies^46^ have shown that chronic neuropathy correlates with the severity of acute sensory neuropathy, raising the possibility that reduction in acute symptoms could also provide protection from chronic neuropathy.

## Discussion

Tyrosine phosphorylation is a key regulatory mechanism of intra- and intercellular signalling in metazoans^47^. Recent global phosphoproteomic analysis^30^,^31^,^48^,^49^ using high-throughput, high-sensitivity MS have revealed an unexpectedly complex repertoire of proteins that can be tyrosine phosphorylated. Extensive tyrosine phosphorylation in secreted or extracellular proteins was one such unexpected observation, which later led to the identification of VLK (vertebrate lonesome kinase) as a secreted tyrosine kinase that acts in the extracellular environment^50^. Analysis of these global phosphoproteome^30^,^31^ data have now revealed that membrane transporters in general, but ‘multispecific' drug transporters in particular are tyrosine phosphorylated. Functional studies showed that many important SLC-family drug transporters^8^ (OCT1, OCT2, OCT3, MATE1 and OATP1B1) might be regulated through tyrosine phosphorylation. This regulatory mechanism was explored in depth for OCT2, which led to the identification of the Src-family kinase Yes1 as the kinase responsible for OCT2 tyrosine phosphorylation both *in vitro* and *in vivo*.

Identification of tyrosine phosphorylation as a crucial mechanism of drug transporter regulation has potentially wide ranging implications. Drug–drug interactions or genetic polymorphisms that alter transporter function are known to greatly affect therapeutic outcomes, including efficacy as well as toxicities^9^. Our study suggests that deregulation of post-translational modifications of drug transporters might have similar pharmacological consequences. We show that TKIs can be a potential cause of such deregulation. Tyrosine kinases are major targets of not only currently used drugs, but also of ongoing drug discovery programs^18^. Interestingly, several clinically used TKIs are known to modulate drug transporter functions^28^,^51^,^52^. Some of these interactions could be competitive, while, in case of ATP-binding cassette transporters, TKIs could occupy ATP-binding sites. However, as we show here for OCT2, TKIs can also inhibit drug transporters by targeting tyrosine kinases essential for their function. Multiple TKIs that target Yes1 and other potent Yes1 inhibitors like dorsomorphin inhibited OCT2 function. Importantly, TKI-resistant Yes1 was able to rescue OCT2 inhibition, providing compelling evidence in support of our hypothesis.

The Yes1 kinase is a member of Src family of non-receptor tyrosine kinases (SFK) involved in diverse cellular processes^53^. Among SFKs, Src, Fyn and Yes1 are expressed in a variety of cell types, while the rest are restricted to haematopoietic cells^54^. Yes1 is highly expressed in the epithelial cells of the kidney, intestine, liver and lung^55^. While Yes1-deficient mice do not show any overt phenotype, they have significantly reduced transcytosis of polymeric immunoglobulin A across epithelial cells^56^. Our study suggests that through OCT2 phosphorylation, Yes1 may also regulate the transport of organic cations across epithelial cells. Interestingly, Yes1 has been implicated in the phosphorylation of occludin^57^, which is essential for epithelial tight junction maintenance. Higher Yes1 expression in epithelial cells, its role in transcytosis of macromolecules and tight junction maintenance, combined with our identification of its role in OCT2 regulation raises the possibility that among the SFKs, Yes1 might have functionally diverged to regulate transport processes across epithelia.

The clinically used Yes1 inhibitor dasatinib^27^ reduced OCT2 function in both cell culture and *ex vivo* models. Importantly, we carried out proof-of-principle experiments to determine the *in vivo* effect of pharmacological or genetic inhibition of Yes1 on OCT2 function at two major sites the kidneys and DRGs. These studies showed that renal OCT2 phosphorylation and function was significantly reduced by Yes1 inhibition *in vivo*. Similarly, dasatinib-mediated Yes1 inhibition or genetic Yes1 knockdown reduced OCT2 phosphorylation and function in the DRGs. These studies utilizing two models, which provide an *in vivo* readout of OCT2 function, suggest that Yes1 is essential for OCT2 phosphorylation and function *in vivo*. These studies also suggest that Yes1 could be pharmacologically targeted to reduce OCT2 function. OCT2 inhibition has the potential to mitigate acute sensory neuropathy^19^, a debilitating and common side effect of chemotherapy^58^, which occurs, in part, due to OCT2-mediated oxaliplatin uptake in the DRGs. However, the clinical translation of these findings would require in-depth studies to determine the effect of combining oxaliplatin and dasatinib on both the anti-cancer efficacy and acute as well as chronic neuropathy.

Collectively, in the current study, we have uncovered the underappreciated role of post-translational regulation of drug transporters. These drug transporters are highly expressed in intestine, liver and kidney, major tissues that regulate absorption, distribution, metabolism and excretion of drugs, including TKIs. Due to TKI accumulation in these tissues, drug transporters are uniquely vulnerable to phosphorylation-mediated interaction with TKIs. We propose that widely used therapeutics that target protein kinases, either due to inhibition of ‘on-target' or ‘off-target' protein kinases, can alter post-translational modifications of drug transporters and possibly drug-metabolizing enzymes and ion channels, which has widespread pharmacological implications.

## Methods

### Cell culture and reagents

Parental Hela, HEK293 cells were obtained from American Type Culture Collection (ATCC). Hela, HEK293, Hela-OCT2 and HEK293-OCT2 cells were cultured in DMEM supplemented with 10% FBS and grown at 37 °C in a humidified incubator containing 5% CO_2_ as described previously^21^. Lipofectamine 2000 or LTX (Life Technologies) reagent was used for transient transfections, followed by uptake assays, 24 h later. Tyrosine kinase inhibitors were obtained from Sigma-Aldrich or Selleckchem. Radiolabelled compounds were obtained from American Radiochemicals or Moravek Biochemicals.

### Site-directed mutagenesis

The OCT1, OCT2, OCT3, MATE1, OATP1B1, YES1, LYN and KDR plasmids with pCMV6-Entry (C-terminal FLAG tagged) backbone were obtained from Origene. The QuikChange II XL Site-Directed Mutagenesis Kit (Agilent) was utilized to generate mutants, according to suggested methods. The mutagenesis primers were designed using the QuikChange Primer Design program and synthesized by Integrated DNA Technologies. These plasmids were sequenced to confirm successful mutagenesis and then used for transient transfection experiments.

### Protein analysis

Whole-cell lysates from cultured cells and tissues were made in modified RIPA buffer (20 mM Tris-HCl (pH 7.5), 150 mM NaCl, 1 mM Na_2_EDTA, 1 mM EGTA, 1% NP-40, 2.5 mM sodium pyrophosphate, 1 mM beta-glycerophosphate, protease and phosphatase inhibitors) supplemented with 1% SDS. Membrane extracts from cultured cells were prepared using Cell surface protein isolation kit from Pierce (89881), while the Endoplasmic reticulum isolation kit was obtained from Sigma (ER0100). Lysates for immunoprecipitation were made in modified RIPA buffer supplemented with 0.1% SDS. Lysates for co-immunoprecipitation experiments were made in modified RIPA buffer supplemented with 0.2% β-maltoside. Immunoprecipitation was carried out using anti-FLAG (EZview Red ANTI-FLAG M2 Affinity Gel, Sigma) and anti-Phospho-Tyrosine (P-Tyr-1000 Rabbit mAb Sepharose) beads. Invitrogen Bis-tris gradient mini or midi-gels were used for western blot analysis, followed by detection by ECL reagent (Cell Signaling). Primary antibodies used were from cell signaling: FLAG (14793) and Phospho-Tyrosine (8954), Santa Cruz Biotech: Yes1 (8403), Phospho-Yes1 (130182), Na^+^/K^+^ ATPase (sc28800) and β-actin (47778), Alpha diagnostic international: Oct2 (OCT21-A) and Abcam: Transferrin receptor (ab84036) and PDI (ab5484). All primary antibodies were used at 1:1,000 dilution. Secondary antibodies were from Jackson Immunoresearch and used at 1:4,000 dilutions. Uncropped images of western blots are shown in Supplementary Figs. 15–18. The enzyme activity of recombinant DPD was measured as previously described^59^.

### Cellular accumulation studies

Uptake experiments were performed with tetraethylammonium, oxaliplatin, metformin and ASP using standard methods^19^,^24^, in the presence or absence of inhibitors with results normalized to uptake values in cells transfected with an empty vector or DMSO-treated groups. In typical uptake experiments, medium was removed; cells were rinsed with PBS, followed by preincubation with either DMSO or inhibitors for 15 min, followed by addition of substrate and uptake measurement after 10–15 min incubation. Fluorescence measurements were done for ASP, while uptake for other substrates was measured by scintillation counter as described previously^60^.

### Small-molecule HTS for OCT2 inhibitors

HEK293 cells stably overexpressing OCT2 were used for optimizing the assay conditions for HTS of OCT2 inhibitors. Briefly, for the primary screen, cells were plated at 3,500 cells per well in 25 μl medium per well (384-well black clear bottom plates, tissue culture treated) and on day 3, compounds were transferred (final test compound at 28 μM concentration and positive control was 140 μM imipramine) along with OCT2 substrate ASP at 5.6 μM and incubated for 10 min, followed by three wash cycles and fluorescence measurement (excitation 492 nm/emission 590 nm). Data analysis and positive hit selection was done according to standard methods. Compounds that inhibited OCT2 activity by >90% were considered as positive hits. TKIs, which were selected for further investigation, were then validated in secondary screens using standard uptake assays.

### siRNA kinome screening

HEK293-OCT2 cells were used for the siRNA kinome screening using methods similar to a previous study^61^. Briefly, the Dharmacon human siRNA library targeting protein kinases and related genes (total 779 genes) and containing four pooled siRNAs for each gene was utilized for this purpose. Briefly, the HEK293-OCT2 cells were plated in 384-well plates and reverse transfected with 25 nM siRNA using Lipofectamine RNAiMAX reagent (Life Technologies). At 48 h post-transfection, cells were incubated with using 10 μM ASP (4-4-[4-(dimethylamino)styryl]-*N*-methylpyridinium iodide) followed by fluorescence readout of OCT2 function. This was followed by CellTiter-Glo Luminescent Cell Viability Assay (Promega). The siRNAs that reduced OCT2 function to ≤75% of control siRNA without affecting cell viability were selected for secondary screen. Deconvoluted secondary screen were performed by methods similar to the primary screen. In a separate secondary screen, Hela-OCT2 cells were reverse transfected with pooled siRNA (Sigma) and plated in 24-well plates, followed by uptake assays with 2 μM TEA after 48 h.

### Single-molecule imaging

Single-molecule imaging on X. laevis oocytes was performed after 1 day of expression at 18 degrees using Total Internal Reflection Fluorescence Microscopy (TIRFM) set-up as described elsewhere^32^,^33^,^62^. Briefly, oocytes were manually devitellinized and placed on high refractive index coverglass (*n*=1.78) and imaged using Olympus 100X, numerical aperture 1.65 oil immersion objective at room temperature. mEGFP-tagged OCT2 (at C terminus) was excited using a phoxX 488 (60 mW) laser. Six hundred frames at the rate of 20 Hz were acquired for subunit counting. Only single, immobile and diffraction-limited spots were analysed. The number of bleaching steps was determined manually for each single spot included in the analysis. The error bars in subunit-counting data show statistical uncertainty in counting and are given by √*n*, where *n*=number of counts. In addition, SiMPull was performed on whole oocyte or only plasma membrane as described previously. Briefly, channels/flow chambers were prepared on coverslips passivated with monofunctional and biotinylated polyethylene glycol. Biotinylated anti-EGFP antibody (Abcam) was then immobilized by incubating 40 nM of antibody on Neutravidin (Thermofisher) coated channels. Sample lysate was flown through the channels and TIRFM imaging and single-molecule counting analysis was done as described above.

### OCT2 structural modelling

Human OCT2 (SLC22A2; accession number O15244) was modelled based on the 2.9 Å resolution X-ray structure of a high-affinity phosphate transporter PiPT (PDB ID 4J05)^34^. The template was selected because of the shared MFS fold assignment structure quality, and sequence similarity to OCT2. The sequence alignment was obtained by manually refining the combined output from PROMALS3D^63^ and MUSCLE^64^ servers. One hundred models were generated using the ‘automodel' class of MODELLER 9.13 (ref. 65) and subsequently assessed by the normalized discrete optimized protein energy (zDOPE)^66^ potential and the protein orientation-dependent statistically optimized atomic potential (SOAP-Protein-OD)^67^. The top scoring models were used for mapping possible phosphorylation sites. The ligand was copied from the PiPT structure as a rigid body for illustrative purposes only.

### Yes1 kinase assay

Yes1 recombinant human protein was obtained from Life technologies (A15557). To purify OCT2 proteins, FLAG-tagged wild-type or mutant OCT2 constructs (described in the site-directed mutagenesis section) were subcloned into pT7CFE1-CHis plasmid (Thermo Fischer). These constructs were then used for *in vitro* translation using a HeLa cell lysate-based Kit (1-Step Human Coupled IVT Kit – DNA; 88881, Life Technologies). The *in vitro* translated proteins were then purified using His Pur cobalt spin columns (Thermo Scientific). For *in vitro* kinase assays, recombinant Yes1 and purified OCT2 proteins were incubated in a kinase buffer (Cell Signaling, 9802) supplemented with cold ATP (Cell signaling, 9804) at 30 °C for 30 min. After the incubation period, the reaction was terminated and OCT2 proteins were immunoprecipitated by FLAG tagged beads (described in the Protein Analysis section) followed by western blot analysis to determine OCT2 tyrosine phosphorylation.

### Animal experiments

Yes1-deficient mice (129S7 background, stock no. 002280) were obtained from Jackson laboratories and Oct1/2-deficient mice (FVB background, model no. 6622) were obtained from Taconic and heterozygous mice were bred in-house to obtain wild-type and knock-out littermates. For some experiments, wild-type FVB mice were obtained from Taconic. For all experiments, age matched (8–12 week) male mice were used. Animals were housed in a temperature-controlled environment with a 12 hour light cycle and given a standard diet and water *ad libitum*. All animals were housed and handled in accordance with the Institutional Animal Care and Use Committee of St Jude Children's Research Hospital or approved by a governmental committee overseeing animal welfare at the University of Münster and performed in accordance with national animal protection laws. For TEA experiments, [^14^C]-TEA (0.2 mg kg^−1^) was injected intravenously followed by plasma collection 5 min later. Urinary bladder, renal and liver tissues were also collected and tissue or plasma TEA levels were measured by scintillation counter. Similar experiments were carried out after dasatinib (15 mg kg^−1^ oral gavage) administration and TEA injection (intravenous), 30 min later. For hydrodynamic injection, siRNAs from Ambion (25 μg in 0.5 ml of PBS) or 0.5 ml of PBS was rapidly injected into the tail vein. After 48 h, [^14^C]-TEA (0.2 mg kg^−1^) was injected followed by plasma and tissue collection. Plasma TEA levels were used to determine OCT2 function, while renal tissues were processed for western blot analysis to determine Yes1 knockdown and OCT2 tyrosine phosphorylation. The *ex vivo* renal tubule uptake assays were performed as described previously^43^,^68^. For pharmacokinetic analyses, male wild-type or Oct1/2^−/−^ mice were administered oxaliplatin (i.p. 10–40 mg kg^−1^) and/or dasatinib (oral gavage 15 mg kg^−1^). Plasma was collected at various time intervals. Urine was collected from animals housed in metabolic cages for 72 h after oxaliplatin administration. Oxaliplatin levels were analysed by flameless atomic absorption spectrometry^19^ and dasatinib levels were measured by LC-MS/MS as described previously^69^.

### Mice DRG isolation

L4 DRGs were extracted from male 8- to 12-week-old mice as reported previously^19^. For whole-cell lysate preparation and immunoprecipitation experiments, DRGs from two mice were pooled, lysed in RIPA buffer, followed by standard immunoprecipitation and western blot analysis. For primary satellite cell culture, the extracted DRGs were further processed according to a modified protocol based on a previous study^70^. Briefly, DRGs from two mice were collected in a 1.5-ml tube containing 500 μl of ice-cold PBS without Ca^2+^ and Mg^2+^, supplemented with antibiotics and D-glucose (6 mg ml^−1^). DRGs were then digested in 500 μl of 5 mg ml^−1^ collagenase for 20-60 min at 37 °C; followed by addition of 100 μl of 0.25% trypsin, in the last 10 min of incubation. At the end of the incubation, 600 μl DMEM (10% FBS and antibiotics) media was added and DRGs were mechanically dissociated using a 1-ml tip and transferred to a 25-cm^2^ flask (with 10 ml medium), and incubated at 37 °C for 2–3 h. At the end of this preplating time, neurons floating in the incubation media were removed by discarding the media, while satellite cells remained attached to the flask and were supplemented with fresh medium. After 4–7 days, when the cells reached confluence, they were detached with trypsin-EDTA and resuspended in fresh medium, and plated at a density of 100,000 cells per ml in six-well plates for further experiments.

### Mouse models of oxaliplatin neuropathy

The mouse models of oxaliplatin neuropathy have been previously reported^19^. Thermal sensitivity associated with a single i.p. oxaliplatin dose (10 mg kg^−1^) in male wild-type and Oct1/2^−/−^ mice was assessed by a cold-plate test. The number of paw lifts and licks when exposed to a temperature of −4 °C for 5 min was obtained for each mouse at 24 h before receiving oxaliplatin to determine the baseline levels. Data were recorded as the percentage change in the number of paw lifts or paw licks compared with baseline values when the animals were exposed to the same temperature 24 h. post-oxaliplatin administration. Mechanical allodynia was determined by a Von Frey Hairs test as described previously. Paw withdrawal was assessed in triplicate on each hind paw with 5-min intervals. Data were recorded as the per cent change of force (in g) necessary to promote paw withdrawal before and after oxaliplatin administration. Investigators conducting the experiments were blinded to the mouse genotypes. To determine the effect of dasatinib treatment on oxaliplatin neuropathy, male wild-type FVB mice were injected with dasatinib (oral gavage), followed by oxaliplatin injection intra-peritoneal 30 min later. Cold-plate assay and Von Frey Hairs test were then performed as described above.

### Statistical considerations

Data are presented as mean with s.e., unless stated otherwise. Statistical calculations (Student's *t*-test or analysis of variance) were done using a Graph-pad Prism. *P*<0.05 was considered statistically significant.

## Additional information

**How to cite this article:** Sprowl, J. A. *et al*. A phosphotyrosine switch regulates organic cation transporters. *Nat. Commun.* 7:10880 doi: 10.1038/ncomms10880 (2016).

## Supplementary Material

Supplementary Information Supplementary Figures 1-18.

Supplementary Data 1 Data analysis of small molecule high throughput screening of OCT2 inhibitors.

Supplementary Data 2 Summary of positive hits obtained from Primary siRNA Kinome screen.

Supplementary Data 3 List of phosphorylation sites in pharmacological important proteins obtained from Phosphosite.
