# Supplementary material for: A phosphotyrosine switch regulates organic cation transporters
Source: Nat Commun. 2016 Mar 16;7:10880. doi: 10.1038/ncomms10880 (PMC4799362; doi:10.1038/ncomms10880)
Supplement: Supplementary Information — Supplementary Figures 1-18. [file ncomms10880-s1.pdf]

Supplementary Figure 1

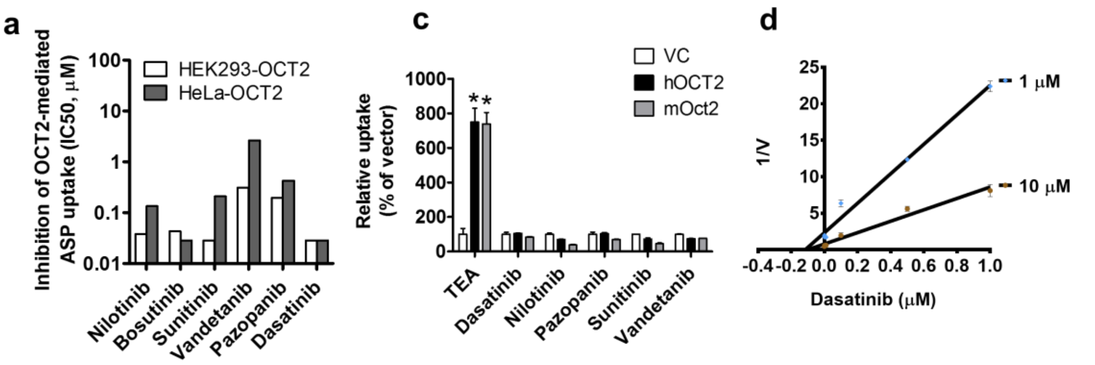

b

TKI	Reference to increased plasma creatinine in prescribing information or IB
Axitinib	Creatinine increased in less than 10% of patients (no grade 3/4)
Bosutinib	Increased serum creatinine in 10% of patients
Crizotinib	N/a
Dasatinib	Grade 3/4 elevated creatinine in 8% of patients
Erlotinib	N/a
Gefitinib	N/a
Imatinib	Increased creatinine rare (1.5% of patients)
Lapatinib	N/a
Nilotinib	Elevated creatinine in <1% of patients
Pazopanib	Creatinine increased in 26% (any grade) of patients (Rautiola et al, Acta Oncol 2014)
Ponatinib	Creatinine increased in 7% (any grade) and <1% of patients (grade 3/4)
Regorafenib	N/a
Ruxolitinib	N/a
Sorafenib	N/a
Sunitinib	Abnormal creatinine in 12% (any grade) and 1% of patients (grade 3/4)
Vandetanib	Increased creatinine in 16% of patients (any grade)
Vemurafenib	Grade 3 increases in creatinine more common in females than males

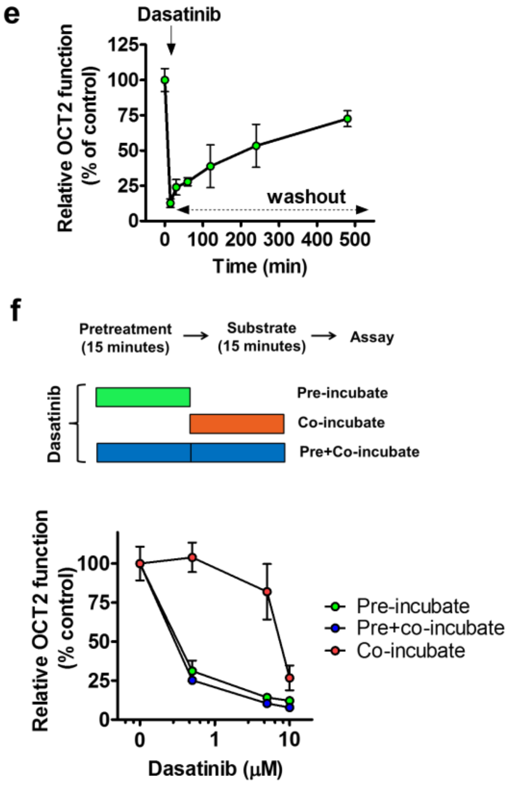

Supplementary Figure 1: TKIs as OCT2 inhibitors. (**a**) IC50 values of OCT2 inhibition in two different cell lines overexpressing OCT2. (**b**) List of TKIs that are known to cause increased serum creatinine levels, which could be a result of OCT2 inhibition. TKIs that have potent OCT2 inhibition activity are highlighted in red. (**c**) HEK293 cells stably expressing vector, hOCT2 or mOct2 were incubated with indicated radiolabeled compounds (2 µM) and cellular accumulation (15 min) was measured by scintillation counter. The graph represents relative uptake values as compared to individual vector group, after normalization of protein levels. * indicates statistically significant as compared to vector (VC) group. (**d**) HEK293-OCT2 cells were used for uptake assays ([^{14}C]-TEA for 15 minutes) at two indicated TEA concentrations and in the presence of 0-1µM dasatinib. The graph depicts a Dixon plot. (**e**) HEK293-OCT2 cells were treated with 10 µM dasatinib for 15 min. Then the drug was washed out, and TEA uptake assays were carried out at different time points between 0 and 8 hrs. Baseline OCT2 activity was determined in untreated cells at each time point. (**f**) HEK293-OCT2 cells were either pretreated with dasatinib for 15 min, followed by TEA uptake assay in the absence of dasatinib (Pre-incubate group) or pretreated with DMSO, followed by TEA uptake assay in the presence of dasatinib (Co-incubate group) or pretreated with dasatinib, followed by TEA uptake assay in the presence of dasatinib (Pre+Co-incubate group).

Supplementary Figure 2

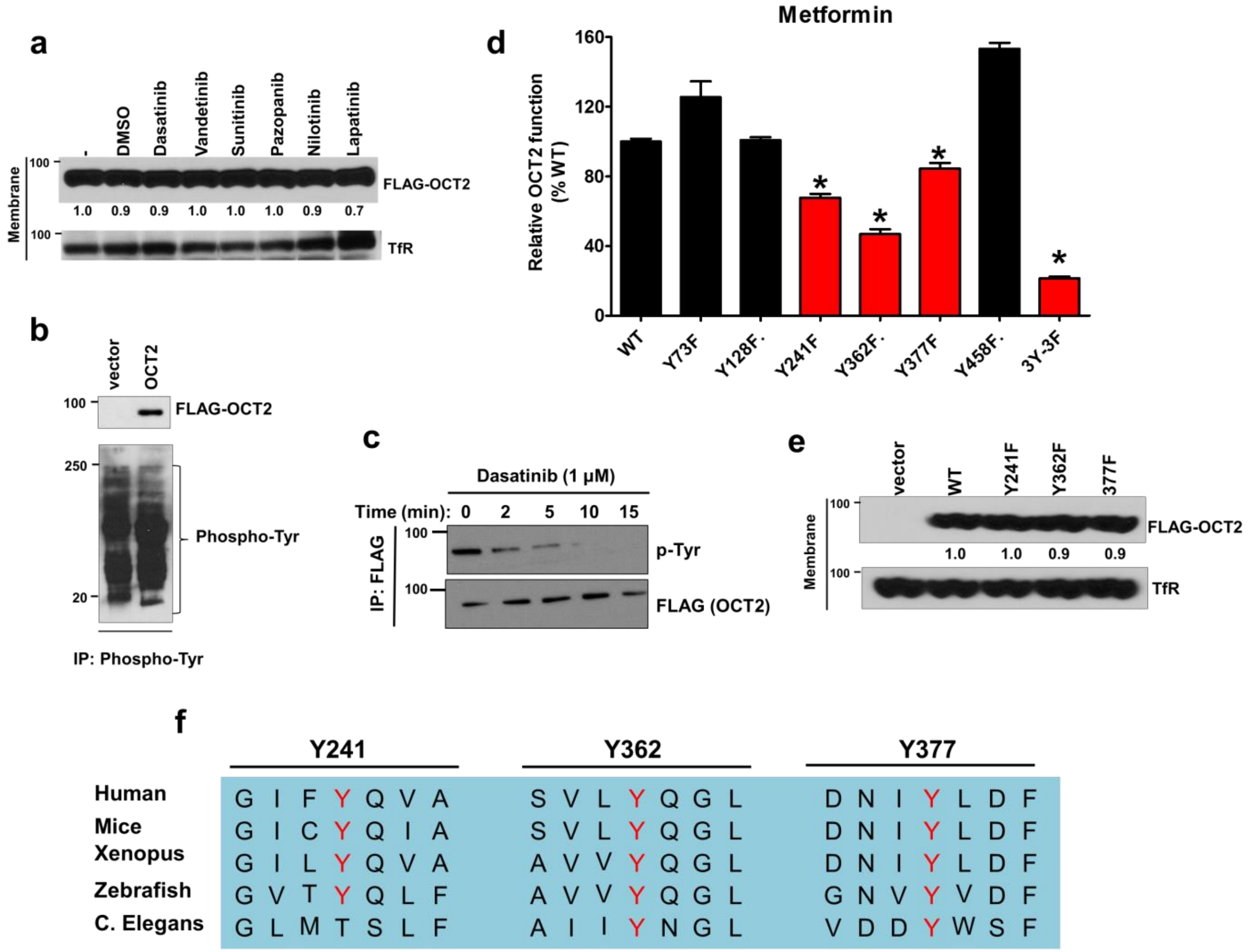

Supplementary Figure 2: Regulation of OCT2 tyrosine phosphorylation. (**a**) Hela-OCT2 cells were treated with either DMSO or indicated TKIs (10 µM) for 30 min followed by membrane extraction and western blot analysis. Transferrin receptor (TfR) served as loading control. (**b**) Hela-vector or Hela-OCT2 cells were lysed and a phospho-tyrosine antibody was used for immuno-precipitation followed by western blot analysis. (**c**) Hela-OCT2 cells were treated with 1µM dasatinib for indicated time points, followed by immunoprecipitation of FLAG-OCT2 and western blot analysis of tyrosine phosphorylated OCT2. (**d**) Hela cells were transfected with indicated wild-type or OCT2 mutants and 24 hours later 50 µM metformin was used for uptake assays (15 min). The graph represents relative uptake as compared to WT group. * indicates statistically significant as compared to WT group. (**e**) Hela cells were transfected with vector, WT or mutant OCT2, followed by membrane preparation 24 hours after transfection. Transferrin receptor (TfR) served as loading control. (**f**) The OCT2 protein sequence from indicated organisms was aligned by a multiple a sequence alignment program (MAFFT).

Supplementary Figure 3

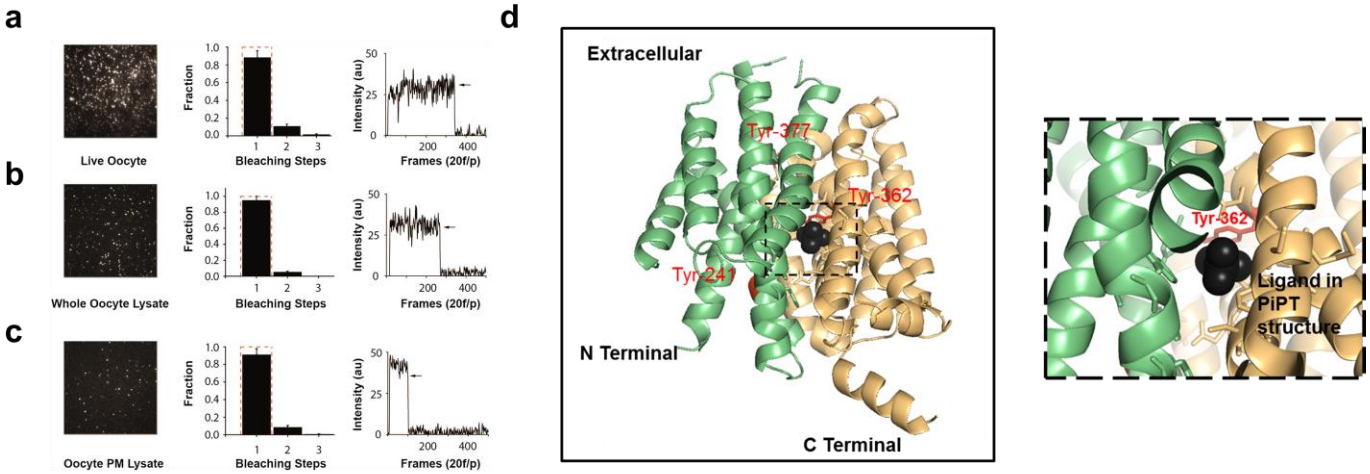

Supplementary Figure 3: Relevance of OCT2 tyrosine phosphorylation. (**a-c**) Single-molecule subunit counting of organic cation transporter 2 Single-molecule irreversible photobleaching to count the number of mEGFPs per fluorescent spot (i.e., number of subunits per transporter) of OCT2 in live Xenopus oocyte plasma membrane (**a**), Using whole oocyte lysate in SiMPull (**b**) and Using oocyte plasma membrane lysate only in SiMPull (**c**). (Left) Images show first frame of the movie to indicate density of spots. (Middle) Average frequency distributions of number of bleaching steps (black bars) with error bars indicating SEM. Dashed red line indicates theoretical binominal distribution for monomer. (Right) Fluorescence traces from single spots showing single step of photobleaching. (**d**) OCT2 structural model showing the three proposed phospho-tyrosine sites and the predicted substrate binding site.

Supplementary Figure 4

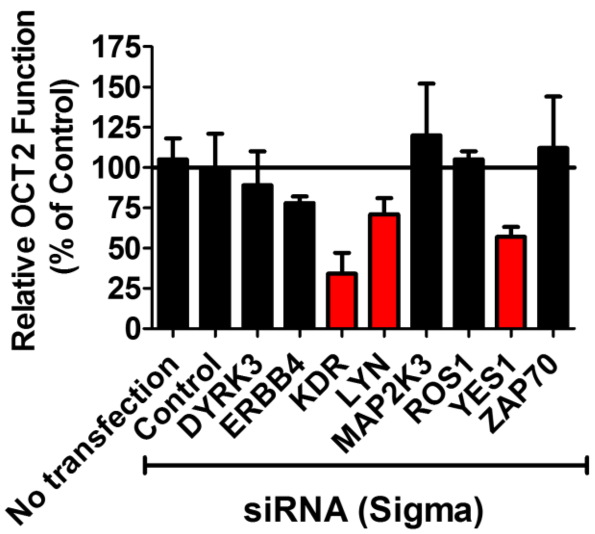

Supplementary Figure 4: Secondary kinase screen to identify OCT2 phoshorylating protein kinases. Hela-OCT2 cells were reverse transfected with 25 nM siRNA (Sigma, pooled) for indicated protein kinases and 72 hours later, OCT2 functional assays were performed using TEA as a substrate (2 µM, 15 min). The graph represents relative uptake as compared to control siRNA transfected group. An arbitrary cut-off of ≤ 75% activity as compared to Control group was used to further short list candidate kinases (KDR, LYN and Yes1) that significantly affected OCT2 function.

Supplementary Figure 5

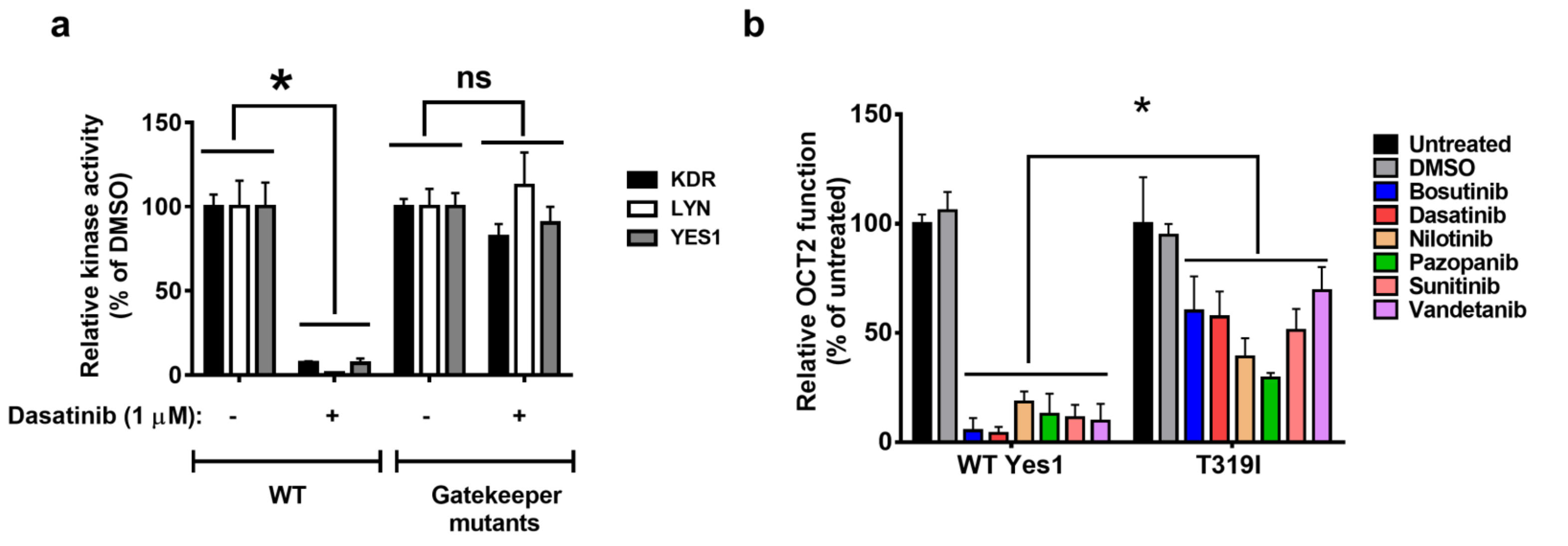

Supplementary Figure 5: Effect of selected TKI resistant Kinase mutants on OCT2 function. (**a**) Hela cells were transfected with WT and gatekeeper mutants of KDR, LYN and YES1. These cells were then treated with either DMSO (-) or 1μM Dasatinib for 15 min, followed by FLAG mediated immunoprecipitation of WT or gatekeeper kinases. The immunoprecipitates were then used for an in vitro kinase assay using Myelin Basic Protein (MBP) as a substrate (30 min, 30°C, ^{32}P ATP). SDS-PAGE gels were run and relative kinase activity was calculated based on the densitometric analysis. * indicates statistically significant as compared to DMSO group (**b**) Hela-OCT2 cells were transfected with either wild type or T319I gatekeeper mutant, followed by pre-treatment with indicated TKIs (1μM and 15 minutes). The pre-treatment was followed by uptake assays (15 minutes) using [^{14}C]-TEA (2μM). * indicates statistically significant as compared to WT Yes1 treated groups.

Supplementary Figure 6

a

TKI	Yes1 K_d (nM)	OCT2 Inhibition
Bosutinib	4	Yes
Dasatinib	0.3	Yes
Erlotinib	2200	Yes
Lapatinib	>10,000	No
Nilotinib	1100	Yes
Pazopanib	5000	Yes
Sunitinib	120	Yes
Sorafenib	>10,000	No
Vandetinib	120	Yes

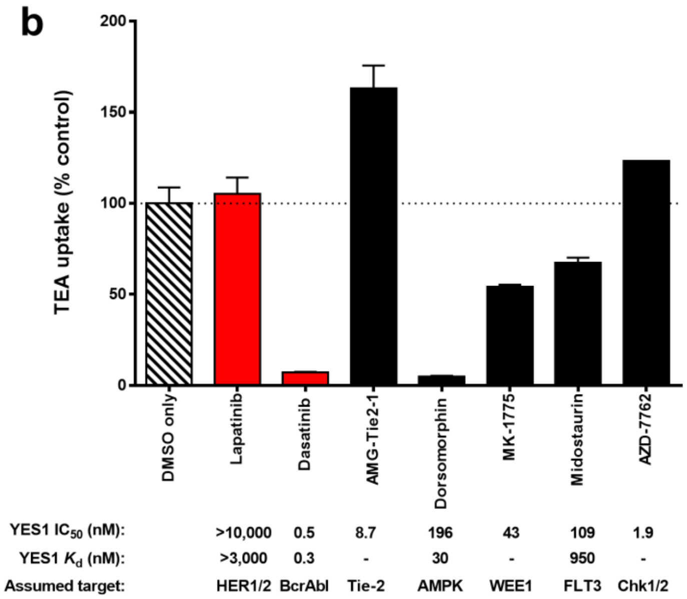

Supplementary Figure 6: Small molecule Yes1 inhibitors reduce OCT2 function. (**a**) TKIs that inhibit OCT2 are also Yes1 inhibitors (based on Davis, M. I. et al. Nat Biotech, 2011 29(11): 1046-1051.). (**b**) Hela-OCT2 cells were pre-treated (1 µM) with indicated compounds including Yes1 inhibitors followed by OCT2 uptake assays using TEA as substrate (2 µM, 15 min). The data is presented as relative OCT2 activity as compared to DMSO group. Hyphen (-) in the lower panel indicates that Kd values are unknown.

Supplementary Figure 7

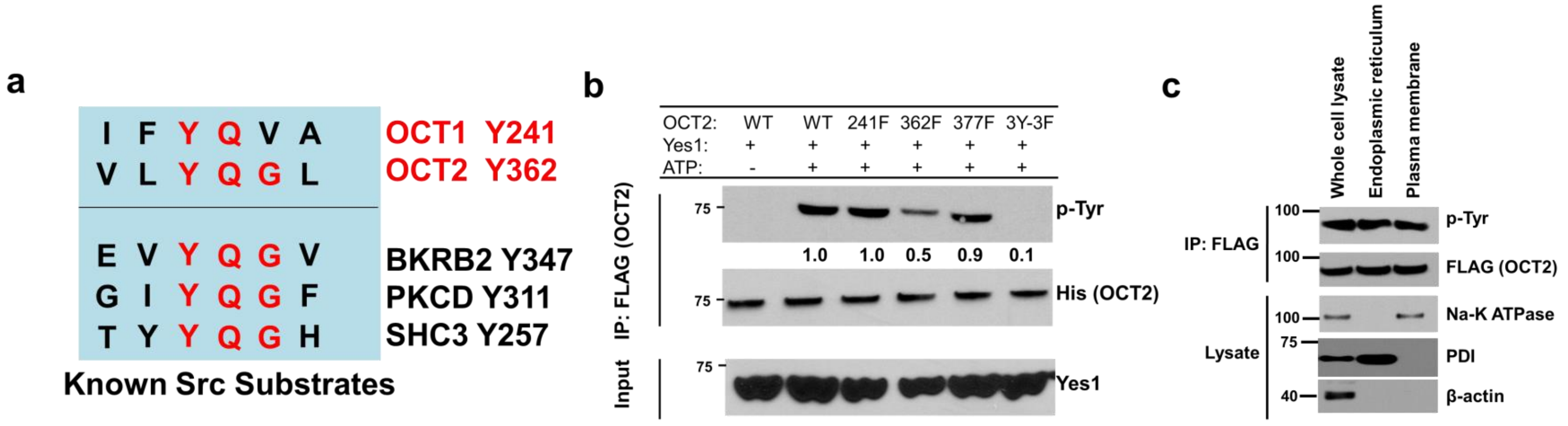

Supplementary Figure 7: Yes1 can phosphorylate OCT2 (**a**) The OCT2 protein sequence surrounding the Y241 and Y362 sites was aligned with other known Src family kinase substrates (PhosphoSite). Multiple Src family substrates had sites similar to sequence surrounding Y241 and Y362 sites in OCT2. (**b**) Purified FLAG-OCT2 wild-type or Y/F mutants and Yes1 were used in a kinase assay in the presence or absence of ATP, followed by His-OCT2-IP and western blot analysis of OCT2 tyrosine phosphorylation and determination of total OCT2 and Yes1 protein levels. (**c**) Indicated subcellular fraction were isolated from Hela-OCT2 cells, followed by FLAG-IP of OCT2 and western blot analysis. The lower panel shows the blots from the indicated lysates and Na-K ATPase (plasma membrane), Protein disulfide isomerase (PDI) (Endoplasmic reticulum) and β-actin (cytosol) were used to check the purity of the isolated fractions.

Supplementary Figure 8

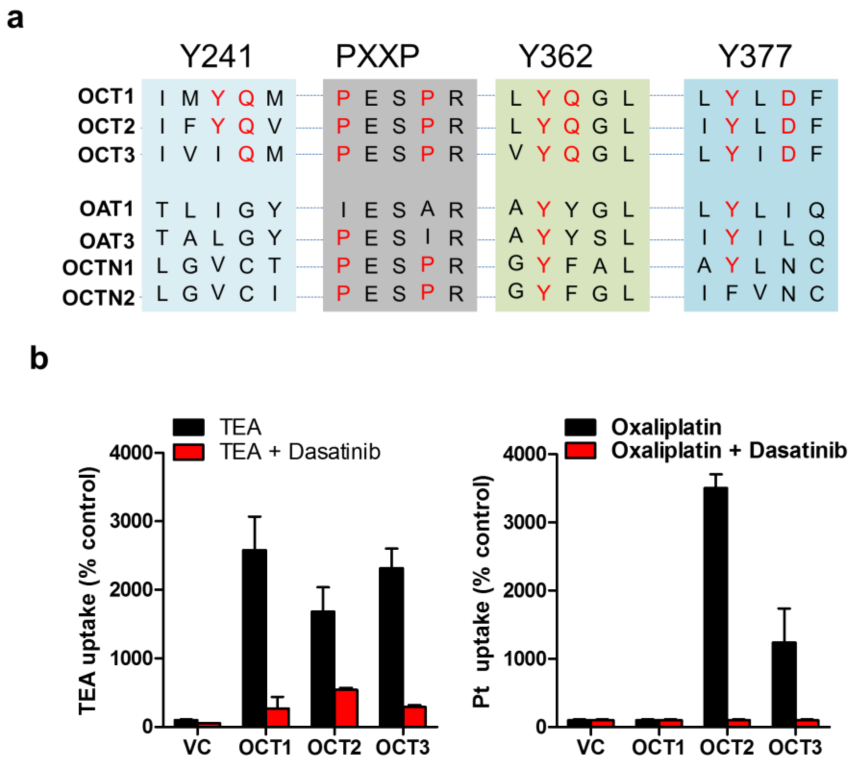

Supplementary Figure 8: Tyrosine phosphorylation sites are conserved in OCTs (**a**) The protein sequence of indicated human organic cation and anion transporters of SLC22 family proteins was aligned by a multiple sequence alignment program (MAFFT). (**b**) HEK293 stably expressing OCT1, OCT2 and OCT3 were used for uptake assays (SLC22A -TEA and Oxaliplatin) in the presence of 10 µM dasatinib. The graphs represent relative transporter activity for indicated substrates as compared to vector (VC) group.

Supplementary Figure 9

a

Drug Transporter	Phospho-Tyrosine site(s)
OCT1	361
OCT2	241
MATE1	299
OAT2	252
OCTN2	486
OATP1B1	367,422,425,625,645
OATP4C1	473, 543
PGP	187
MRP1	277,490,920,1508,1522
MRP2	616,885
MRP3	229,231,720
MRP4	45,1255,1259
MRP5	10,17,1166,1202,1423
BCRP	464

b

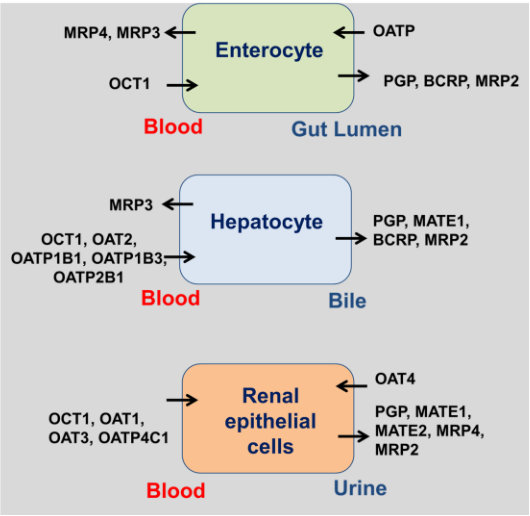

Supplementary Figure 9: Drug-transporters and tyrosine phosphorylation. (**a**) Analysis of high throughput global phosphorylation data (PhosphoSite) suggests that multiple drug-transporters have phospho-tyrosine sites. (**b**) Representative figure showing the localization of important drug-transporters.

Supplementary Figure 10

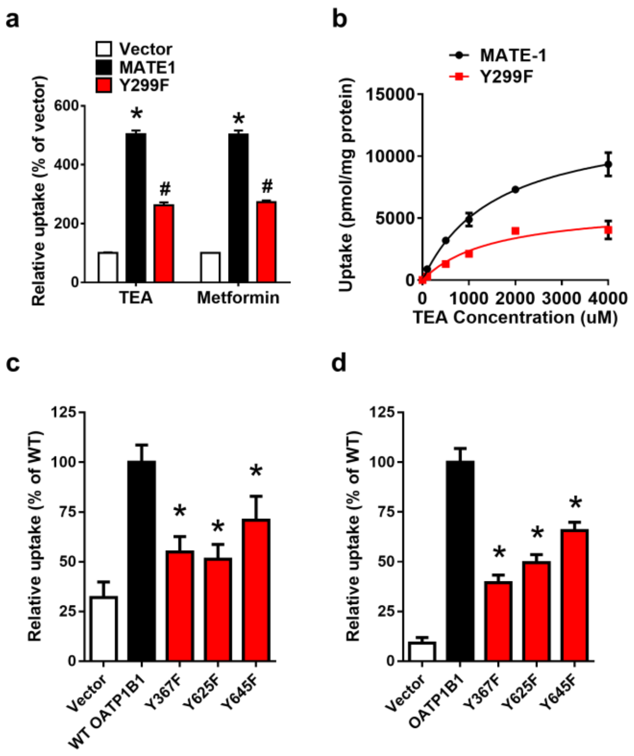

Supplementary Figure 10: MATE1 and OATP1B1 mutants that lack phospho-tyrosine sites have reduced activity. (**a-b**) HEK293 were transfected with indicated MATE1 constructs and uptake assays (15 min incubation) were performed using 2 μM TEA and 50 μM metformin as substrates (**c-d**) HEK293 were transfected with indicated OATP1B1constructs and uptake assays were performed using estradiol-17β-d-glucuronide (E2G) (0.1 μM; 5 min incubation) (**c**) and 8-fluorescein-cAMP (8-FcA) (5 μM; 10 min incubation) (**d**) as substrates. Graphs represent relative uptake activity as compared to WT group. * indicates statistically significant as compared to WT group.

Supplementary Figure 11

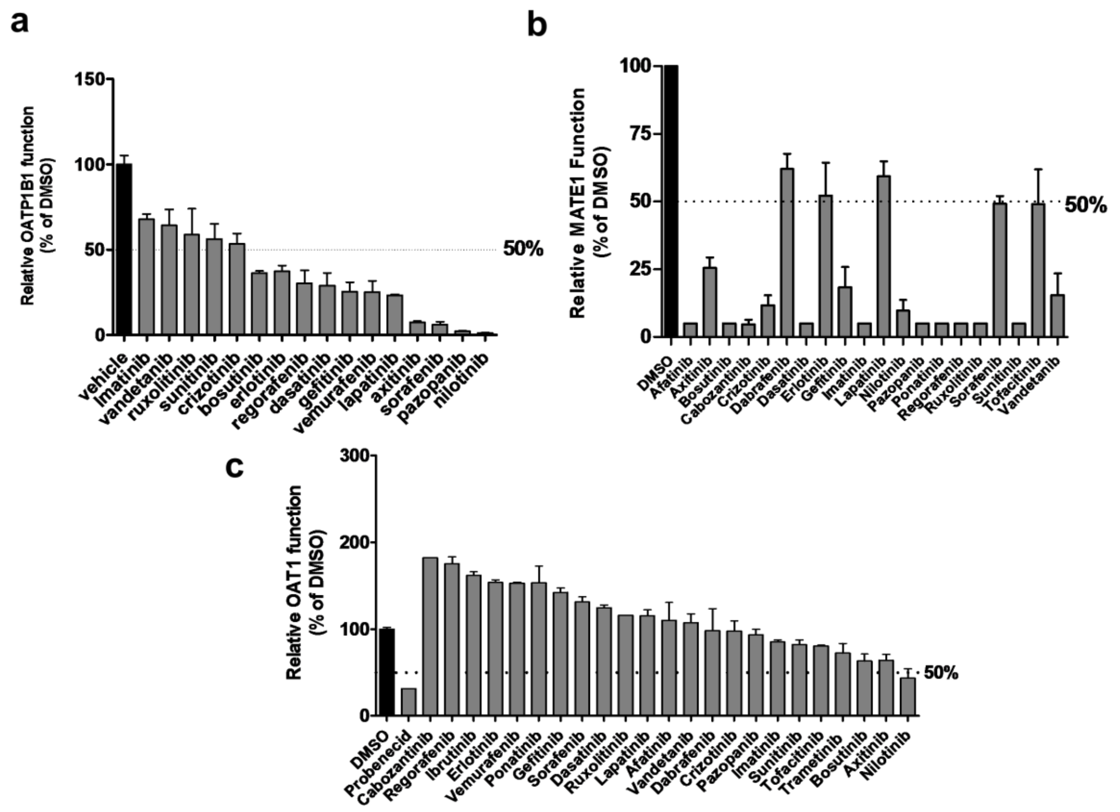

Supplementary Figure 11: Distinct TKI inhibit multiple SLC transporters. HEK293 cells expressing indicated transporters were pre-incubated for 15 min with 10 µM TKIs, followed by uptake assays using indicated substrates for (**a**) OATP1B1 (0.1 µM estradiol-17β-d-glucuronide (E2G) for 5 min) (**b**) MATE1 (2 µM TEA for 15 min) and (**c**) OAT1 (5µM para-aminohippurate (PAH) for 15 min).

Supplementary Figure 12

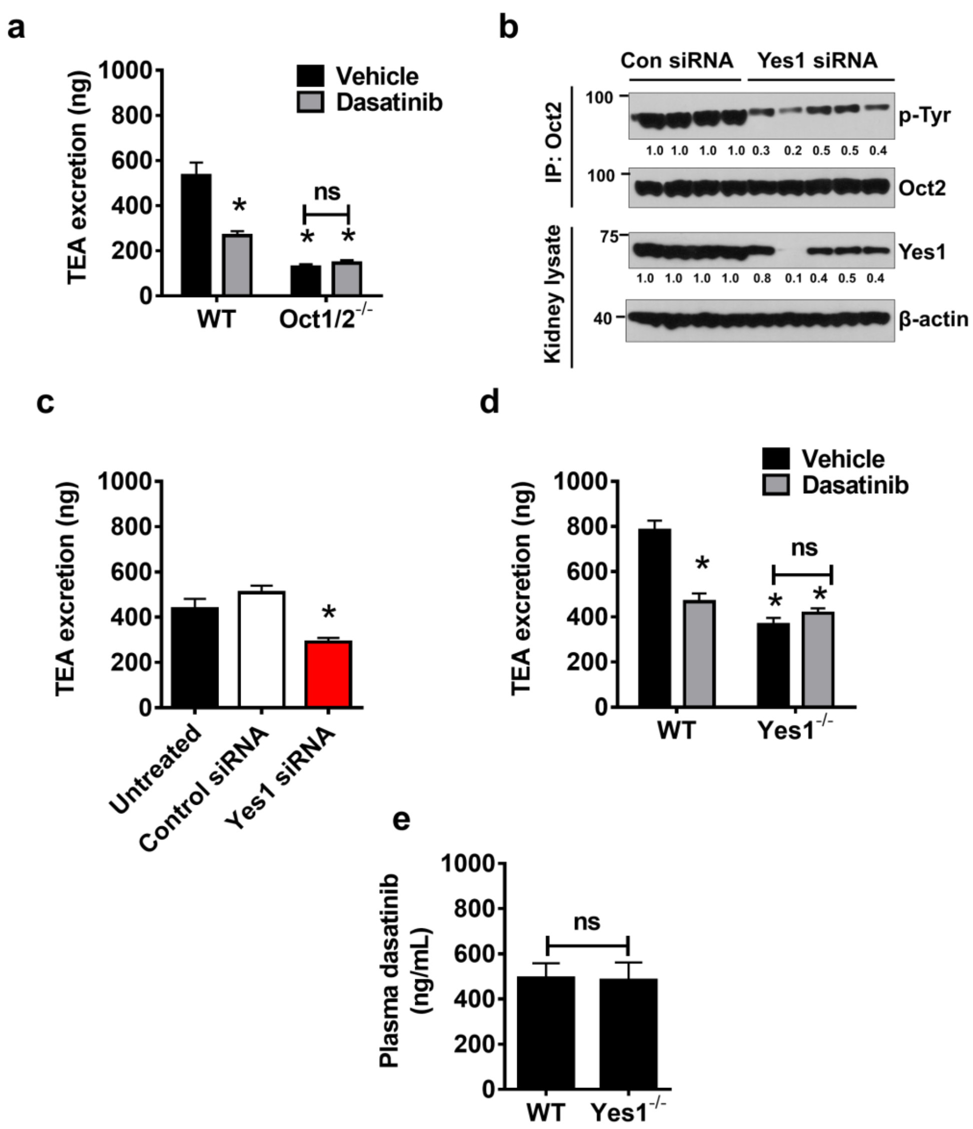

Supplementary Figure 12: Effect of genetic or pharmacological Yes1 inhibition on OCT2 function and phosphorylation *in vivo*. (a) Wild type and Oct1/2$^{-/-}$ mice were injected with either vehicle or dasatinib (15 mg kg^{-1}, p.o.) and 30 min later they were injected with 0.2 mg kg^{-1} C^{14}-TEA (i.v.), followed by urine collection at 5 min. The graph represents urine TEA levels from n=5 mice per group. * indicates statistically significant as compared to WT vehicle group (**b**) FVB mice were injected with control or Yes1 siRNA and 3 days later, kidneys were collected followed by OCT2 immuno-precipitation and OCT2 phospho-tyrosine levels were detected by western blot analysis. Yes1 and actin levels were determined in total cellular lysates. (**c**) Wild type FVB mice were injected with either control or Yes1 siRNA by hydrodynamic tail-vein injection (25 µg in 0.5 ml of PBS). Three days later, the mice were injected i.v. with a 0.2 mg kg^{-1} dose of [^{14}C]-TEA, and urine levels of TEA were measured at 5 min. * indicates statistically significant as compared to untreated group (**d**) Wild type and Yes1$^{-/-}$ mice were injected with 0.2 mg kg^{-1} [^{14}C]-TEA (i.v.) and urine levels of TEA were measured at 5 minutes. * indicates statistically significant as compared to WT vehicle (**e**) Wild type and Yes1$^{-/-}$ mice were injected with dasatinib (15 mg kg^{-1}, p.o.) and 30 min, plasma was collected for dasatinib measurement.

Supplementary Figure 13

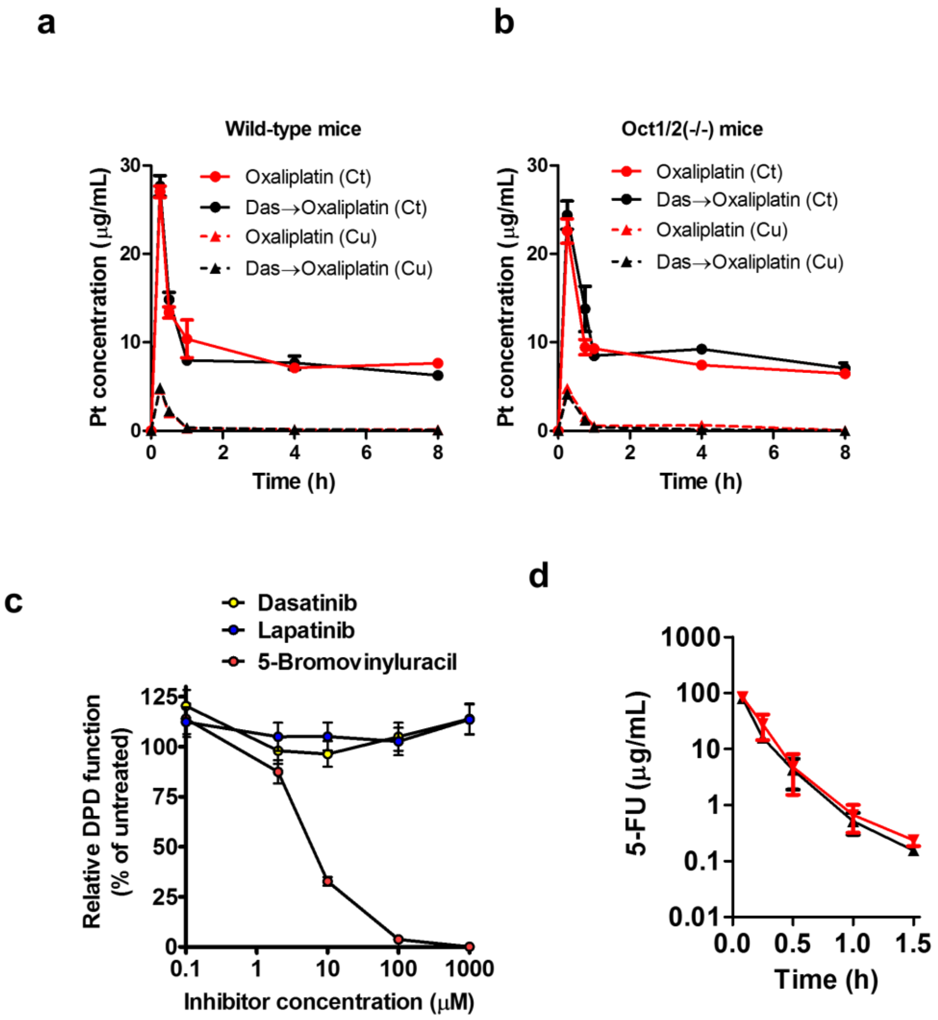

Supplementary Figure 13: Pharmacokinetic analysis of dasatinib, oxaliplatin and fluorouracil (5-FU). (**a**) Plasma concentration-time profiles of total platinum (Ct) and unbound platinum (Cu) in wild type mice following oxaliplatin with and without dasatinib (10 mg kg^{-1}, p.o.). (**b**) Plasma concentration-time profiles of total platinum (Ct) and unbound platinum (Cu) in Oct1/$2^{-/-}$ mice following oxaliplatin with and without dasatinib (10 mg kg^{-1}, p.o.).(**c**) DPD enzyme activity was measured in the presence of the indicated concentrations of dasatinib, lapatinib and 5-bromovinyluracil, a known DPD inhibitor (**d**) Plasma-concentration time profiles of 5-FU (75 mg kg^{-1}, i.v.) in wild type mice in the presence (red) or absence (black) of dasatinib (mg kg^{-1} p.o.; 15 min before 5-FU).

Supplementary Figure 14

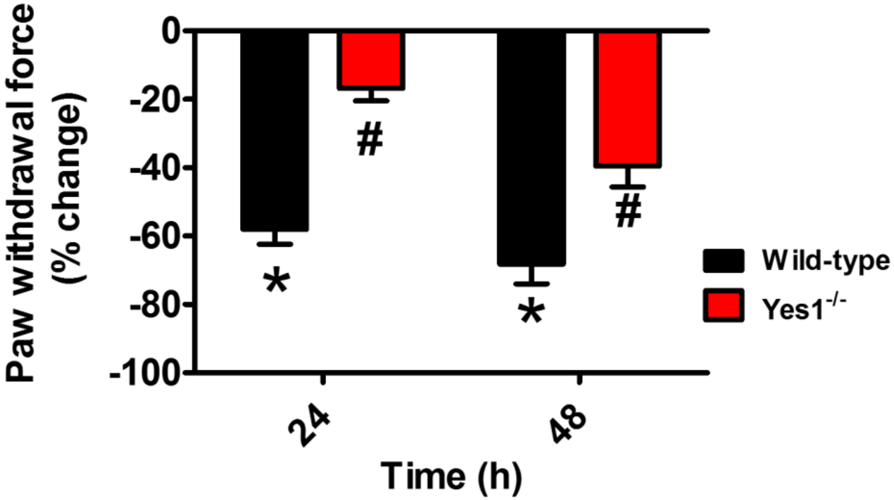

Supplementary Figure 14: Yes1-deficient mice are protected from oxaliplatin neurotoxicity. Mechanical allodynia associated with a single dose of oxaliplatin (40 mg kg^{-1}) in wild type and Yes1 deficient mice as determined by a Von Frey Hairs test. The force required to induce paw withdrawal in grams (g) at baseline was measured following 24 h after drug administration. The graph represents relative percentage change in paw withdrawal force as compared to baseline values. * indicates a statistically significant difference as compared with the baseline (untreated) values.

Supplementary Figure 15

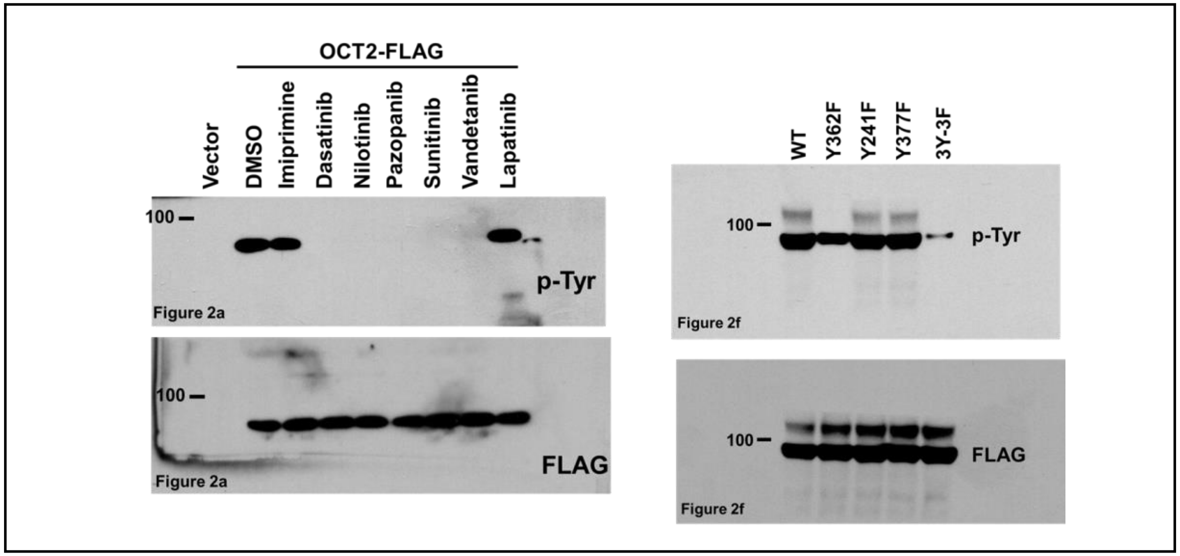

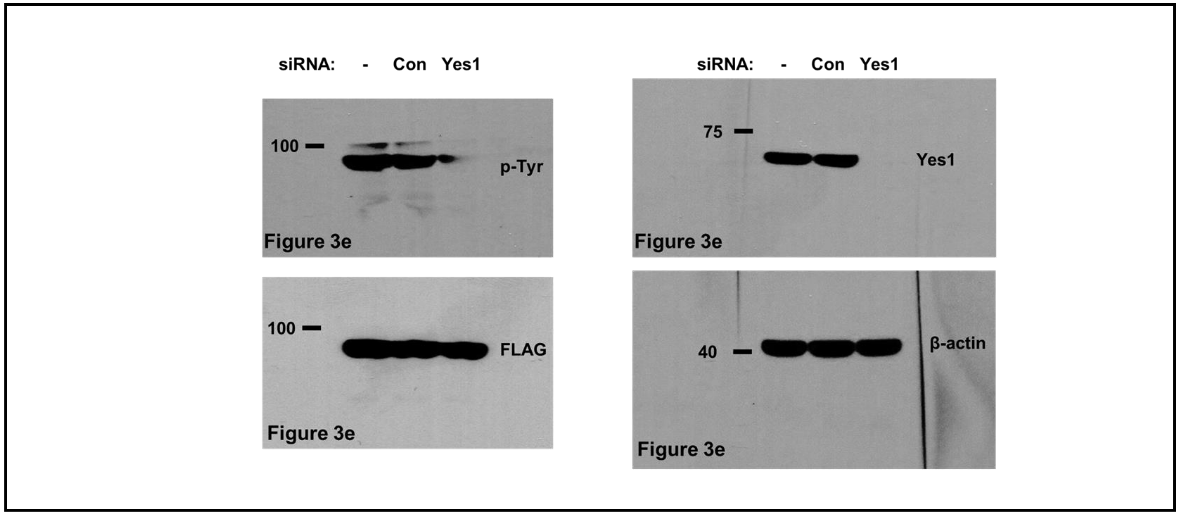

Supplementary Figure 15: Uncropped images of blots shown in Figure 2 (upper) and Figure 3 (lower).

Supplementary Figure 16

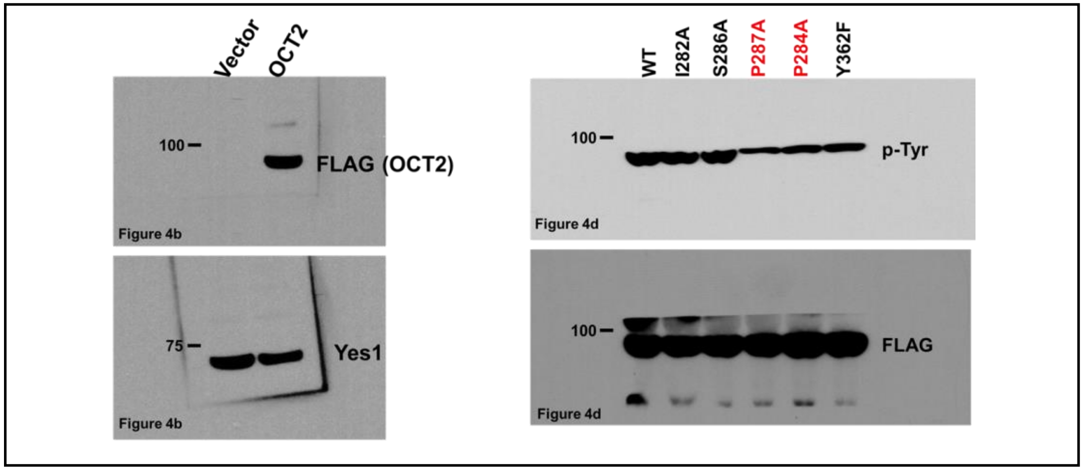

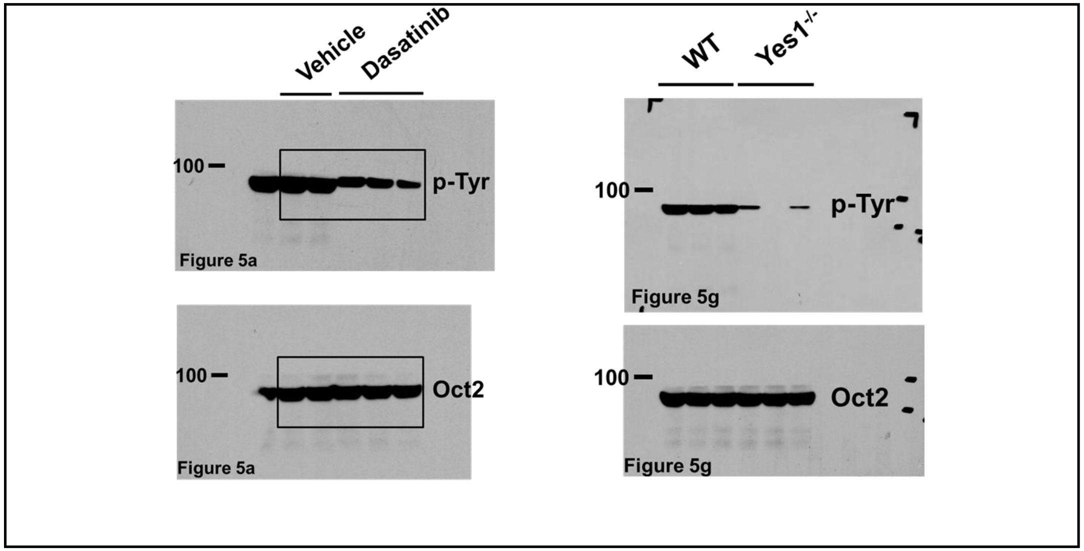

Supplementary Figure 16: Uncropped images of blots shown in Figure 4 (upper) and Figure 5 (lower).

Supplementary Figure 17

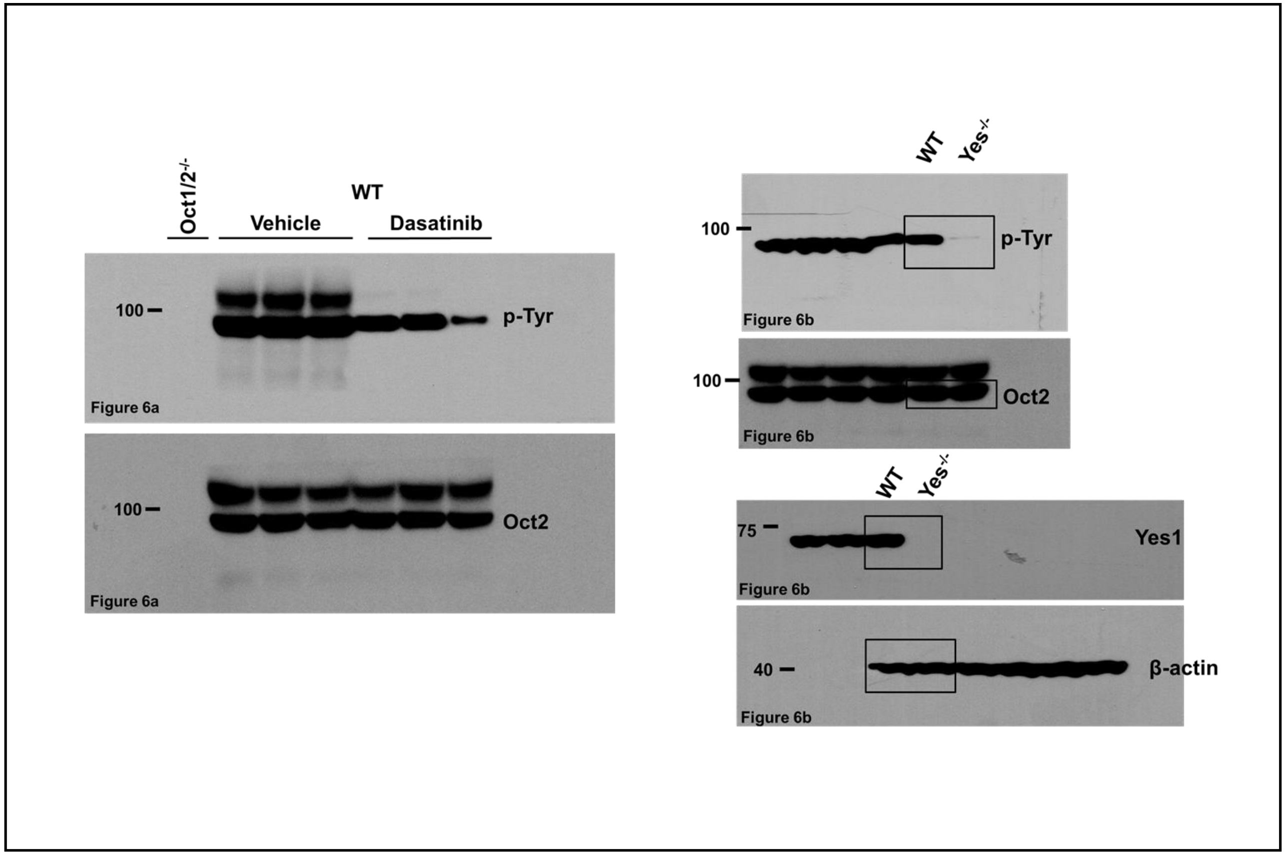

Supplementary Figure 17: Uncropped images of blots shown in Figure 6.

Supplementary Figure 18

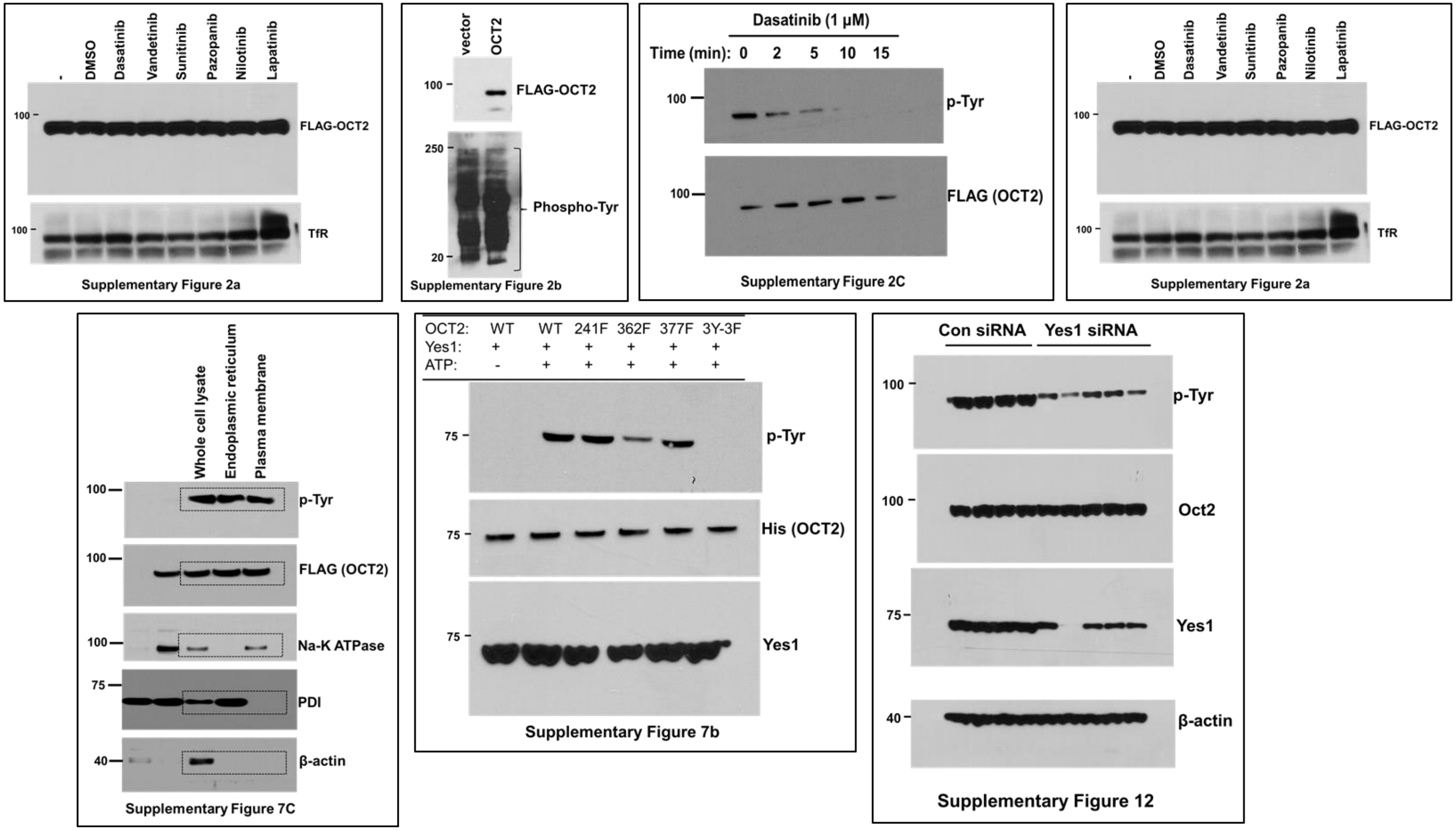

Supplementary Figure 18: Uncropped images of blots shown in Supplementary Figures.
